# A Novel Virulence Strategy for *Pseudomonas aeruginosa* Mediated by an Autotransporter with Arginine-Specific Aminopeptidase Activity

**DOI:** 10.1371/journal.ppat.1002854

**Published:** 2012-08-23

**Authors:** Jeni C. A. Luckett, Owen Darch, Chase Watters, Manal AbuOun, Victoria Wright, Esteban Paredes-Osses, Jenny Ward, Hana Goto, Stephan Heeb, Stéphanie Pommier, Kendra P. Rumbaugh, Miguel Cámara, Kim R. Hardie

**Affiliations:** 1 School of Molecular Medical Sciences, Centre for Biomolecular Sciences, University of Nottingham, University Park, Nottingham, United Kingdom; 2 Department of Surgery, MS 8312, Texas Tech University Health Sciences Center, Lubbock, Texas, United States of America; 3 Department of Bacteriology, Animal Health and Veterinary Laboratories Agency (Weybridge), Addlestone, Surrey, United Kingdom; Massachusetts General Hospital, Harvard Medical School, United States of America

## Abstract

The opportunistic human pathogen, *Pseudomonas aeruginosa*, is a major cause of infections in chronic wounds, burns and the lungs of cystic fibrosis patients. The *P. aeruginosa* genome encodes at least three proteins exhibiting the characteristic three domain structure of autotransporters, but much remains to be understood about the functions of these three proteins and their role in pathogenicity. Autotransporters are the largest family of secreted proteins in Gram-negative bacteria, and those characterised are virulence factors. Here, we demonstrate that the PA0328 autotransporter is a cell-surface tethered, arginine-specific aminopeptidase, and have defined its active site by site directed mutagenesis. Hence, we have assigned PA0328 with the name AaaA, for arginine-specific autotransporter of *P. aeruginosa*. We show that AaaA provides a fitness advantage in environments where the sole source of nitrogen is peptides with an aminoterminal arginine, and that this could be important for establishing an infection, as the lack of AaaA led to attenuation in a mouse chronic wound infection which correlated with lower levels of the cytokines TNFα, IL-1α, KC and COX-2. Consequently AaaA is an important virulence factor playing a significant role in the successful establishment of *P. aeruginosa* infections.

## Author Summary

We present a new *Pseudomonas aeruginosa* virulence factor that promotes chronic skin wound infections. We propose the name AaaA for this cell-surface tethered autotransporter. This arginine-specific aminopeptidase confers a growth advantage upon *P. aeruginosa*, providing a fitness advantage by creating a supply of arginine in chronic wounds where oxygen availability is limited and biofilm formation is involved. To our knowledge, this is the first mechanistic evidence linking the upregulation of genes involved in arginine metabolism with pathogenicity of *P. aeruginosa*, and we propose potential underlying mechanisms. The superbug *P. aeruginosa* is the leading cause of morbidity in cystic fibrosis patients. The ineffective host immune response to bacterial colonization is likely to play a critical role in the demise of these patients, making the possibility that AaaA could interface with the innate immune system, influencing the activity of iNOS and consequently the host's defence against invading pathogens. The surface localisation of AaaA makes it accessible to inhibitors that could reduce growth of *P. aeruginosa* during colonisation and alter biofilm formation, potentially improving the efficacy of current antimicrobials. Indeed, structurally related aminopeptidases play a central role in several disease states (stroke, diabetes, cancer, HIV and neuropsychiatric disorders), and inhibitors alleviate symptoms.

## Introduction

*Pseudomonas aeruginosa* is an important human pathogen causing a myriad of infections including those of burns, trauma wounds and the eyes [1]. This Gram-negative bacterium is perhaps best known for being the leading cause of morbidity in cystic fibrosis (CF) patients, with 80% of adult CF patients carrying *P. aeruginosa* in their lungs [2], [3], and has recently gained notoriety by being classified as a ‘superbug’ by the media. The latter emanates from the intrinsic resistance that this opportunistic pathogen has against antibiotics [4], [5], and its prominence as a cause of nosocomial infections (there are an estimated 10,000 cases each year in UK hospitals) [5]–[8].

The success of *P. aeruginosa* as a pathogen is attributed to the immense battery of virulence determinants that it possesses. These virulence factors include toxins, proteases, lipases, and rhamnolipids [9], which are regulated by a complex hierarchy of regulators that include cell-to-cell communication networks [10]–[12]. One of the least-studied families of virulence factors produced by *P. aeruginosa* is the autotransporters (ATs). ATs are characterized by a tripartite structure encompassing (i) an *N*-terminal signal peptide that enables translocation across the inner membrane, (ii) a *C*-terminal domain that forms a β-barrel in the outer membrane, and (iii) a central passenger domain that bears the functional domain of the AT. ATs have diverse functions, but all of those characterized to date in pathogens contribute to the virulence of their producing bacterium [13], [14]. The functions of ATs include agglutination (e.g. Ag43 [15]), vacuolating toxins (e.g. VacA [16], [17]) and serine proteases. The serine protease ATs of the *Enterobacteriaceae* are termed SPATES, and their functions as we understand them have been comprehensively reviewed [13], [18]. SPATES tend to be the most abundant proteins secreted during *in vitro* growth of pathogenic *Enterobacteriaceae*, and usually exhibit multifunctional virulence-related activities. Other ATs with proteolytic activities include NalP, which processes other ATs and is responsible for the release of the lactoferrin-binding protein B (LpbB) from the surface of *Neisseria meningitidis* [19]. Additional ATs involved in surface maturation of proteins include SphB1 of *Bordetella pertussis* [20] and AasP of *Actinobacillus pleuropneumoniae* [21]. Some ATs use their proteolytic activities to direct their own release via autoproteolysis from the surface of the bacteria that produces them, e.g. IgA protease of *Neisseria gonnorhoeae* [22], and Hap from *Haemophilus influenzae* [23]. In some cases, although the proteolytic AT clearly augments virulence and influences interactions with host cells, its precise role is uncertain, e.g. PfaI from *Pseudomonas fluorescens* [24]. There are also examples where ATs combine proteolysis with other functions to ameliorate pathogenicity, e.g. the *Proteus mirabilis* toxic agglutinin Pta [25].

Whilst ATs appear to have the simplest mechanism of secretion found in Gram-negative bacteria and constitute a subgroup of the type V secretion system, the mechanistic details of AT secretion are currently controversial and need further study [26]. Originally it was proposed that the β-barrel acted as the outer membrane conduit to secrete the passenger domain to the bacterial surface [22]. More recently it has become evident that additional proteins may be involved in this process, most notably the recently discovered Bam complex [27]–[30], but the mechanistic steps are hotly debated [14], [18], [26]–[29], [31]–[33]. Once at the bacterial cell surface, some ATs, such as IgA protease from *Neisseria gonorrohoeae*, release their passenger domains into the extracellular matrix [22], whilst others like the Ag43 produced by enteroaggregative *E. coli* maintain the functional passenger domain on their cell surface [15].

The genome of *P. aeruginosa* encodes a number of proteins with a type V mode of secretion [9], including a recently described member of a novel subgroup (type Vd [34]). However, it only harbours three genes encoding proteins predicted to have the characteristic AT β-barrel domain. One of these, the esterase EstA, functions to alter the levels of extracellular rhamnolipids, modulates twitching, swimming and swarming motility and influences the formation and architecture of biofilms [35], [36]. The isolation of an attenuated mutant indicates that EstA contributes to the virulence of *P. aeruginosa*, although the underlying mechanisms require further investigation [37]. The two other ATs in *P. aeruginosa* are not as well studied. While PA3535 is a putative serine protease [38], there is currently no published experimental evidence to confirm this. Similarly, little is known of the function of PA0328, although it is annotated as an aminopeptidase. The MEROPS database of peptidases predicts PA0328 to be a member of the M28 family of peptidases [39]. The M28 family encompasses both amino and carboxy specific peptidases which tetrahedrally coordinate two zinc ligands using residues including histidine, aspartic acid and glutamic acid to create their catalytic pocket [40]–[43].

Here, we describe the characterisation of PA0328. Since we demonstrate that PA0328 is an AT that specifically removes aminoterminal arginine from peptides, we propose to name it the Arginine-specific Aminopeptidase of *P. aeruginosa*: AaaA. In addition to characterizing the surface localization of AaaA, and its ability to confer a growth fitness advantage, we describe the influence that AaaA has on the pathogenicity of *P. aeruginosa*, and put forward potential mechanistic models for this role.

## Results

### AaaA is an AT that is not released from the bacterial cell surface

**Figure 1:**
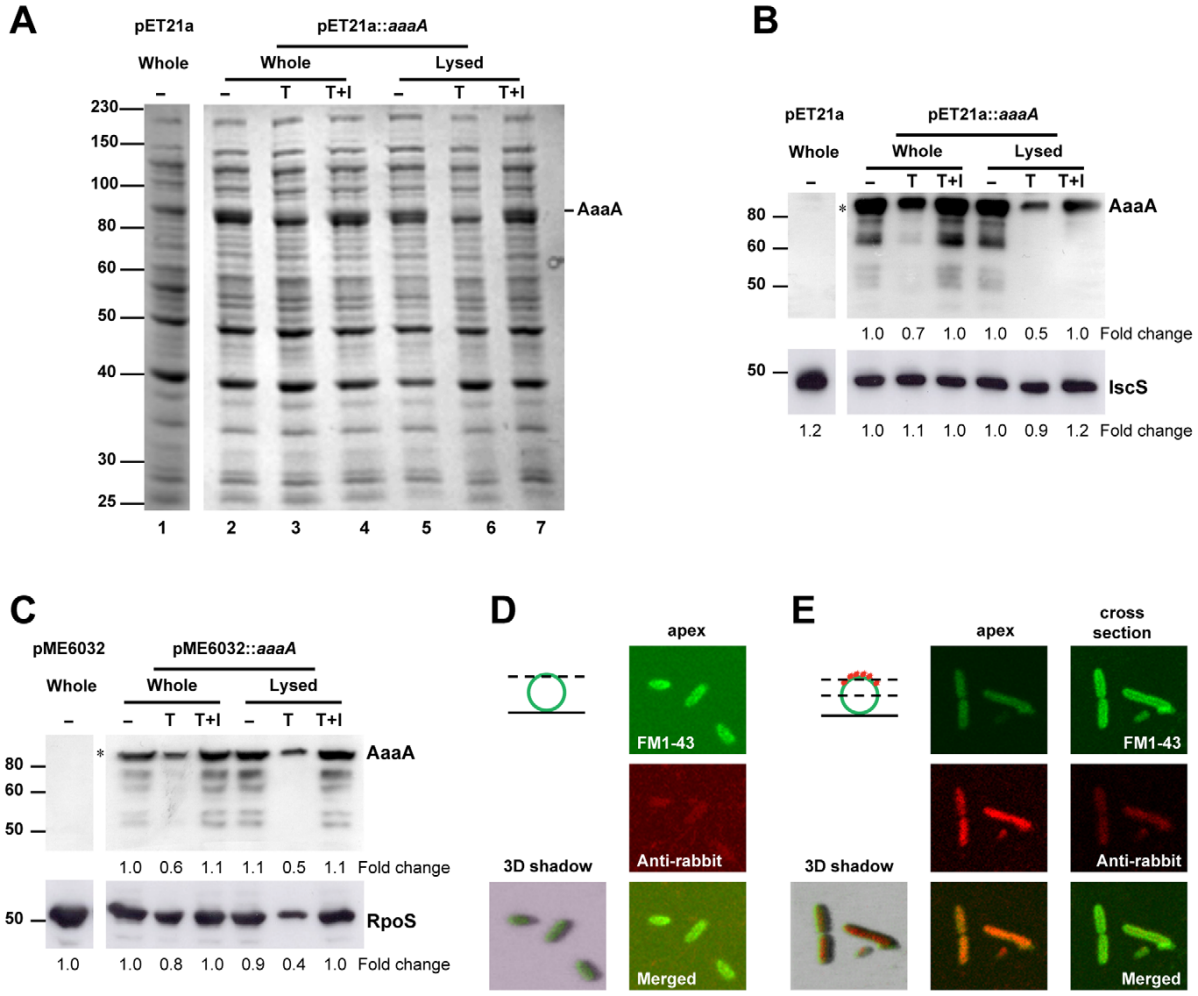
The passenger and β-barrel domains of AaaA remain connected and are tethered to the cell surface. *E. coli* LEMO21 bearing the empty vector pET21a or pET21a::*aaaA* was grown to mid exponential phase in LB, and induced with 1 mM IPTG for 1 h. Following harvesting, washing and resuspension in PBS-Hepes, half of the cells were lysed by sonication. The whole and lysed cells were split into three aliquots and incubated with (T) or without (−) trypsin according to the Materials and Methods. Trypsin inhibitor was added at the same time as trypsin to one of the aliquots (T+I). Proteins were separated through a 9% SDS PAGE and stained with Coomassie Blue (Panel A) or subjected to immunoblotting with either α-AaaA (Panel B, top), or α-IscS (Panel B, bottom) antisera. A parallel experiment was performed with *P. aeruginosa* Δ*aaaA* bearing either pME6032 or pME6032::*aaaA*. LB overnight cultures were diluted 1∶100 in fresh LB, grown for 3 h at 37°C, and induced with 1 mM IPTG for 1 h. The immunoblot of the *P. aeruginosa* proteins is shown in Panel C, with the cytoplasmic control protein being detected with α-RpoS in the bottom panel. The sizes of molecular weight markers are shown in kDa on the left, and the position of AaaA is indicated. In Panels B and C, densitometry was used to estimate the quantity of the cytoplasmic protein and the full length AaaA (indicated with the asterisk) detected in the immunoblots using imageJ software. The fold change of AaaA, IscS and RpoS are shown below the images of the respective immunoblots. The images in Panels D and E were captured by confocal fluorescent microscopy. *P. aeruginosa* Δ*aaaA*(pME6032::*aaaA*) was grown and induced as described for Panel C, probed with FM1-43 and either α-AaaA (Panel E) or pre-immune serum (Panel D). Incubation with donkey α-rabbit alexa fluor 680-conjugated secondary antibody (red) was performed before images were captured at either the apex or cross section of individual cells (as indicated in the dotted lines of the cartoon). Green fluorescence from FM1-43 (top Panel, green circle in cartoon), red fluorescence from alexa fluor 680 (middle Panel, red stars in cartoon), merged 2D and merged 3D shadowed images are shown.

To verify that the predicted AT domain located in the *C*-terminal of AaaA functions to direct it to the outer membrane (OM), and to determine whether there is subsequent proteolytic release of the passenger domain, the cellular localisation of AaaA was first analysed. To do this, *aaaA* was inserted adjacent to an IPTG inducible promoter with a C-terminal His-tag resulting in the plasmid pDEST42::*aaaA*. The protein production profiles observed by SDS-PAGE and immunoblotting with the α-His antibody of this clone expressed in *E. coli* DH5α revealed a protein associated with the bacterial cells with a predicted mass of approximately 85 kDa which is larger than 70.4 kDa predicted from the sequence of the encoding gene. Peptide mass fingerprinting confirmed that this protein was indeed AaaA, lacking its *N*-terminal signal peptide (not shown). Subcloning into pET21a was undertaken as described in materials and methods to enable overexpression of *aaaA* from a T7 promoter (Figure 1A, lane 2). The resultant protein was purified, verified by peptide mass spectroscopy, and used to generate specific polyclonal antisera to aid AaaA detection.

To establish whether AaaA was exposed on the cell surface, the AaaA-specific polyclonal antiserum was used to detect whether exogenously introduced trypsin could degrade AaaA (Figure 1). To enable detection in *P. aeruginosa* as well as *E. coli*, *aaaA* was cloned into the shuttle vector pME6032 (creating pME6032::*aaaA*), introduced into the *P. aeruginosa aaaA*-deficient mutant (Δ*aaaA*; Figure S1) and induced with IPTG. The low copy number of pME6032::*aaaA* in *E. coli* DH5α led to AaaA levels too low to be detected easily by immunoblot. Therefore, AaaA was overproduced from pET21a::*aaaA* in *E. coli* LEMO2, a strain reported to tolerate higher levels of membrane proteins. The cultures were split into aliquots. These aliquots were treated with trypsin with or without a protease inhibitor, followed by immunoblotting with α-His (not shown) or α-AaaA (Figure 1B,C). As controls, cytoplasmic RpoS (for *P. aeruginosa*) or IscS (for *E. coli*) were detected similarly, and found not to be degraded as much as AaaA in the presence of trypsin. When the cells were lysed before treatment commenced, greater degradation of AaaA and the cytoplasmic control protein was observed. The intensity of the fully mature AaaA protein was reduced in both whole and lysed cells. The presence of trypsin inhibitor prevented degradation by trypsin (Figure 1B,C). This suggested to us that the passenger domain of AaaA is anchored to the bacterial cell surface, where it is accessible to trypsin digestion, consistent with it being an uncleaved AT. No released AaaA passenger domain could be detected in culture supernatants.

To visualise this more directly, *P. aeruginosa* Δ*aaaA*(pME6032::*aaaA*) was subjected to confocal immunofluorescent microscopy with α-AaaA, and its localisation was compared with that of FM1-43, a fluorescent marker that interacts with lipid membranes. Whilst no signal was detected using the pre-immune serum, α-AaaA clearly detected AaaA primarily on the surface of *P. aeruginosa* (Figure 1D, E).

**Figure 2:**
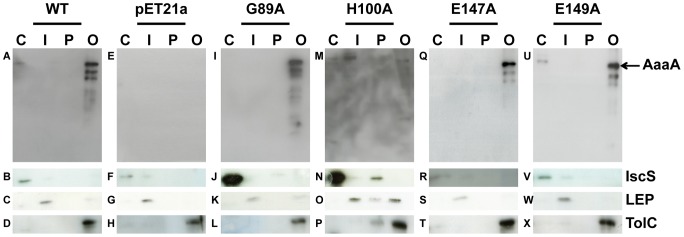
AaaA localises to the outer membrane of *E. coli*. *E. coli* LEMO21 bearing the empty vector (pET21a: **Panels E–H**), pET21a::*aaaA* (WT: **Panels A–D**) or similar plasmids producing one of four site directed mutants (pET21a::*aaaA*_G89A_ (G89A: **Panels I–L**), pET21a::*aaaA*_H100A_ (H100A: **Panels M–P**), pET21a::*aaaA*_E147A_ (E147A: **Panels Q–T**), pET21a::*aaaA*_E149A_ (E149A: **Panels U–X**)) were grown until mid-exponential phase in LB, and induced with 1 mM IPTG for 1 h. Each strain was divided into aliquots to obtain the different fractions of the cell according to the materials and methods (C: cytoplasm; I: inner membrane; P: periplasm; O: outer membrane). Proteins were separated through a 9% SDS PAGE and immunoblotted with α-AaaA (**Panels A,E,I,M,Q,U**), α-IscS (**Panels B,F,J,N,R,V**), α-LEP (**Panels C,G,K,O,S,W**) and α-TolC (**Panels D,H,L,P,T,X**) antisera. The positions of AaaA, IscS, LEP and TolC protein are indicated on the left.

To determine whether the surface exposed AaaA was integrated into the outer membrane, *E. coli* LEMO21(pET21a::*aaaA*) was induced and fractionated. Clean separation of cytoplasmic, periplasmic, inner membrane and outer membrane fractions was verified by immunoblotting with marker proteins: IscS (cystoplasm), LEP (inner membrane), and TolC (outer membrane). Immunoblotting of AaaA revealed that this protein was only detected in the OM (Figure 2A–D).

### AaaA is an arginine-specific aminopeptidase

**Figure 3:**
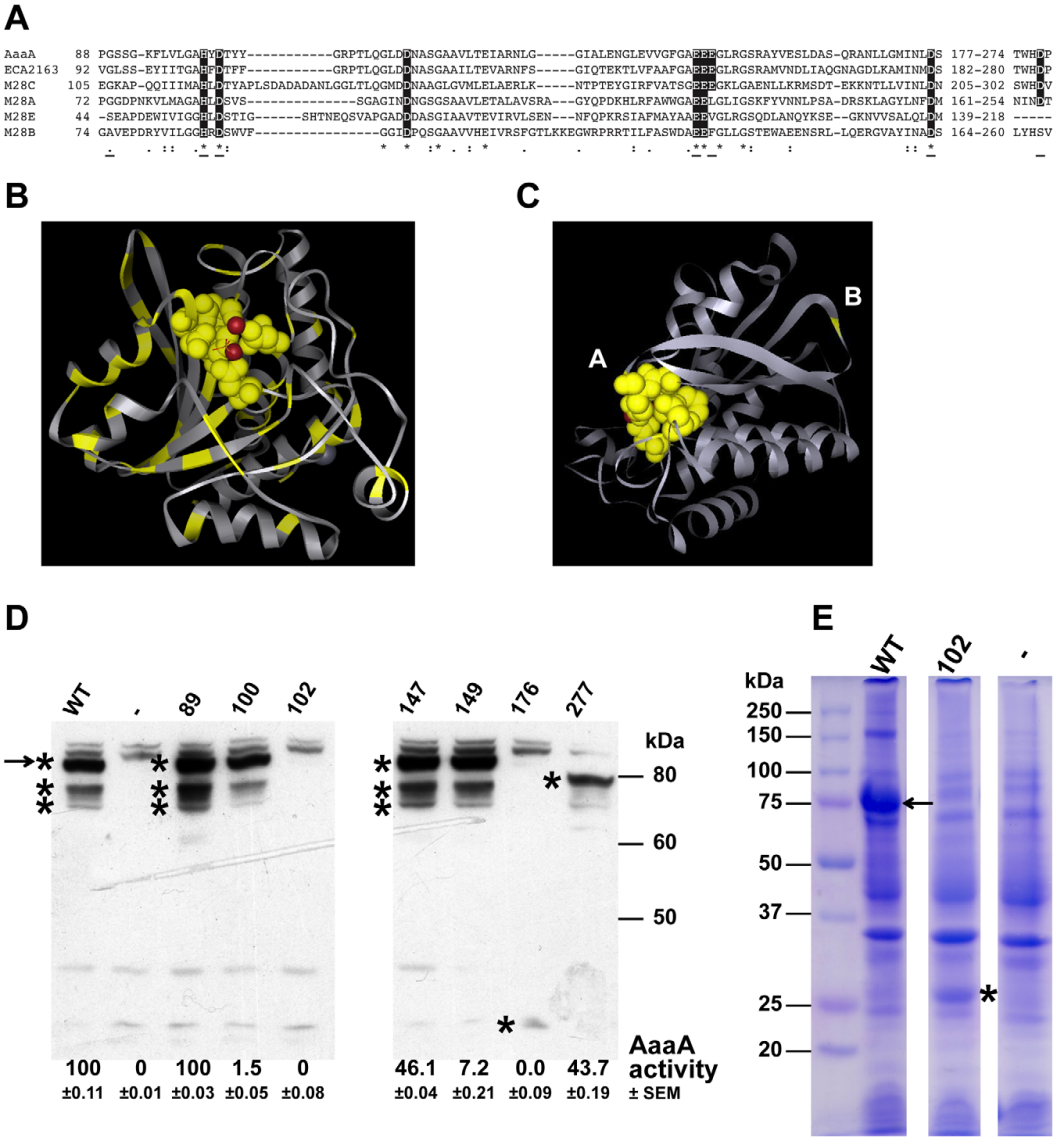
AaaA is a member of the M28 family of aminopeptidases and site directed mutagenesis confirms that predicted active site residues of AaaA contribute to arginine aminopeptidase activity. (**Panel A**) ClustalW2 multiple sequence alignment of the predicted active sites of the holotype enzymes for the four M28 subfamilies plus the two M28C ATs AaaA and ECA2163 (from *Pectobacterium caratovora* subspecies *carotovorum*). Identical residues are indicated by an asterisk, and similar residues by a colon or full stop. The residues highlighted in the black box are those shown to be functional within the active site. Underlining indicates the position of the conserved residues chosen for site directed mutagenesis. The holotype enzymes shown are: *Streptomyces griseus* aminopeptidase S (SGAP) M28.003/MER002161 (M28A), glutamate carboxypeptidase II M28.010/MER002104 (M28B), *E. coli* IAP aminopeptidase M28.05/MER001290 (M28C), and aminopeptidase AP1 M28.002/MER001284 (M28E). All the sequences were taken from UniProt database software (http://www.uniprot.org/). (**Panel B**) Crystal structure of the M28.003 founding aminopeptidase (SGAP) with the residues that are conserved in an alignment with PA0328 highlighted in yellow. The Red balls indicate the two intercalated metal ions. **Panel C** indicates the positions of the residues in AaaA that were selected for mutagenesis. The structure shown was predicted for AaaA using an alignment with and crystal structure of SGAP as the guide. All residues mutated were predicted to be in the active site (A) except G89 which is predicted to lie on an outward facing loop of the protein (B). All mutations were substitutions to Alanine. *E. coli* LEMO21 containing a pET21a vector alone (−) or with WT AaaA or one of the mutated versions (indicated by the mutation) were grown in LB until OD_600_ of 0.5, and induced with IPTG for 3 h. Whole cell extracts were separated through a 9% SDS PAGE and stained with Coomassie Blue (**Panel E**), or subjected to immunoblotting using α-AaaA antibody (**Panel D**). The asterisks indicate products of *aaaA*, and the arrow indicates full length AaaA. The relative activities of each mutant AaaA in the arginine-*p*-nitroanilide assay determined as described in Figure 4 are listed below their respective lane on the immunoblot in Panel D. The activity following incubation of cells with the substrate for 6.5 h is shown as this was the point when wild type AaaA reached maximal absorbance at 405 nm. The absorbance at 405 nm was adjusted to the level of AaaA made in each particular case by dividing by the amount of AaaA quantified from the immunoblot using densitometry performed with the ImagJ software. The standard error of the mean (SEM) for each is also shown.

The passenger domains of ATs characteristically bear the functional active site. Thus, to provide an initial clue towards the function of AaaA, BLAST searches were performed using sequences corresponding to the passenger domain of AaaA. Similarity to aminopeptidase proteins was revealed, confirming the annotation of AaaA [38]. Closer analysis using the MEROPS database (employing a BLAST search of this database using the full length sequence of AaaA), identified AaaA as a member of the M28.005 family. The protein with the closest similarity (63% sequence identity over the entire length of the proteins) was another uncharacterized AT from the plant pathogen *Pectobacterium carotovorum* (ECA2163). Other members of the wider M28 family include both amino and carboxy peptidases with different specificities, the best studied of which being the aminopeptidase A of *Streptomyces griseus* (SGAP) and the leucine-specific aminopeptidase of *Vibrio proteolyticus* (VpAP), which have been crystallized [42]–[48]. Predicted active site catalytic and ligand binding site residues are 100% conserved between AaaA and the other members of the M28.005 family (Figure 3A,B), strongly suggesting that AaaA has an aminopeptidase activity.

*P. aeruginosa* produces a variety of proteases including elastase (LasB), alkaline protease, LasA protease, PrpL, AprA, and a leucine-specific aminopeptidase (paAP; PA2939, [49]). Since the activation of at least one of these (paAP) is mediated by another (elastase), and because the degradation product of one may be the substrate of another, we thought it prudent to investigate the proteolytic function of AaaA in its endogenous background. To do this, an in-frame deletion mutant of *aaaA*, was constructed in PAO1 (see materials and methods, Table 1, Figure S1A).

**Table 1:** Bacterial strains and plasmids used in this study.

Strain/plasmid	Description	Source
***E. coli***		
BL21[DE3]	F^−^ ompT gal dcm lon hsdS_B_(r_B_^−^ m_B_^−^) ë(DE3 [lacI lacUV5-T7 gene 1 ind1 sam7 nin5]) Protein overproduction strain	[118]
DH5α	F^−^ endA1 glnV44 thi-1 recA1 relA1 gyrA96 deoR nupG Ö80d*lacZ*ÄM15 Ä(*lacZYA-argF*)U169, hsdR17(r_K_^−^ m_K_^+^), ë–Cloning strain	[119]
S17-1ë*pir*	recA pro hsdR RP4-2-Tc::Mu-Km::Tn7 tmpR, spcR, strR conjugation strain for suicide vectors	[120]
LEMO21	BL21[DE3] with a fine controllable T7 lysozyme for tunable membrane protein overproduction	[121]
***Pseudomonas aeruginosa***		
PAO1	Nottingham collection wild type strain	[122], [123]
PAO1Ä*aaaA*	In frame deletion of *aaaA* in PAO1	This study
PAJL1	CTX1 inserted into the chromosome of PAO1	This study
PAJL2	CTX1::*aaaA* inserted into the chromosome of PAO1	This study
**Plasmids**		
pBluescript KS+	Cloning vector, ColE1 replicon, Ap^R^	Strategene
pDM4	Suicide vector, *sacBR*, oriR6K, Cm^R^	[111]
pDEST42	Gateway cloning vector, colE1 ori, ApR, T7, LacO	Invitrogen
pET21a	Overexpression plasmid vector, f1 origin, colE1 origin, T7 Promoter, his-tag, lacI, Ap^R^	Novagen
pME6032	pVS1-p15A *E. coli*-*Pseudomonas* shuttle vector, *lacP*-*Ptac* expression vector, Tc^R^	[124]
pminiCTX1	Mini CTX1 vector for construction of chromosomal complementation. Tc^R^	[112]
pDEST42::*aaaA*	Produces AaaA with a C-terminal Histidine tag from pDEST42	Elise Termine and Alain Filloux
pME6032::*aaaA*	Produces AaaA with a C-terminal Histidine tag from pME6032	This study
pBluescript::*aaaA*	*aaaA* cloned into pBluescript KS+	This study
pBluescript::aaaAupstream	600 bp upstream of *aaaA* amplified with primers aaaAfa and aaaArb and cloned between the *Xho*I and *Hind*III sites of pBluescript KS+	This study
pBluescript::aaaAdownstream	600 bp downstream of *aaaA* amplified with primers aaafb and aaaArc and cloned between the *Hind*III and *Spe*I sites of pBluescript KS+	This study
pBluescript::Ä*aaaA*	600 bp flanking *aaaA* with in frame deletion marked by a *Hind*III recognition site, cloned into the *Xho*I and *Spe*I sites of pBluescript KS+	This study
pDM4::Ä*aaaA*	*aaaA* with in frame deletion cloned into *XhoI*/*SpeI* sites of pDM4 from pBluescript::Ä*aaaA*.	This study
pET21a::*aaaA*	*aaaA* cloned into pET21a	This study
pET21a::*aaaA*_G89A_	pET21a::*aaaA* with mutation changing G89 to A	This study
pET21a::*aaaA*_H100A_	pET21a::*aaaA* with mutation changing H100 to A	This study
pET21a::*aaaA*_D102A_	pET21a::*aaaA* with mutation changing D102 to A	This study
pET21a::*aaaA*_E147A_	pET21a::*aaaA* with mutation changing E147 to A	This study
pET21a::*aaaA*_E149A_	pET21a::*aaaA* with mutation changing E149 to A	This study
pET21a::*aaaA*_D176A_	pET21a::*aaaA* with mutation changing D176 to A	This study
pET21a::aaaA_D277A_	pET21a::*aaaA* with mutation changing D277 to A	
pCTX::*aaaA*	pCTX::*lux* containing *aaaA* and the upstream promoter region	This study
**Primers**		
aaaAstart	CTACAGCGACAGCTAATGGTTTGAACACggatcca	This study
aaaAstartNdeI	tatcatatgTTCAAACCATTAGCTGTCGCTG	This study
aaaAstartEcoRI	CAGgaattcGTGTTCAAACCATTAG	This study
aaaAend	gaattcGAACTGCCAGTTCACCCCGAG	This study
aaaAend His	gctATCGATttaGTGATGGTGATGGTGATgGAACTGCCAGTT	For cloning into pME6032, this study
aaaAfa	tatctcgagAGGCCATCGAGTACATCA	This study
aaaArb	ataaagcttCTGGCAGTTCTGAGCG	This study
aaaAfb	tataagcttAATGGTTTGAACACGGCAC	This study
aaaArc	tatactagtATCTGAAGAAAGCGAAAGAC	This study
aaaAminictxFor	GCGGCCGCCGGTGCGCAAGAACTCCCAGC	This study
aaaAminictxRev	GATATCCGCTCAGAACTGCCAGTTCAC	This study
GAPDHfor	AAGGTCGGAGTCAACGGATT	This study
GAPDHrev	TTGATGACAAGCTTCCCGTT	This study
COX-2for	CAGCCAGGCAGCAAATCCT	This study
COX-2rev	ACATTCCCCACGGTTTTGAC	This study
KCfor	ATGGCTGGGATTCACCTCAAG	This study
KCrev	TGAGGGCAACACCTTCAAGG	This study
TNFαfor	ACGGCATGGATCTCAAAGAC	This study
TNFαrev	CGGACTCCGCAAAGTCTAAG	This study
IL-1αfor	CGTCAGGCAGAAGTTTGTCA	This study
IL-1αrev	GTGCACCCGACTTTGTTCTT	This study
AttB1-HIP	ggggacaagtttgtacaaaaaagcaggctccaccgcaggctccaccat	Filloux lab/Invitrogen
AttB2-HIP	GGGGACCACTTTTGTACAAGAAAGCTGGGTAGAAAGCTGGGT	Filloux lab/Invitrogen
*aaaA*::G89Af	CGCCCGCCAGCAGCG	Bases altered to introduce mutation underlined, this study
*aaaA*::G89Ar	CCGAGGCGATGACGTTCTGC	
*aaaA*::H100Af	CTGGTACTCGGCGCGGCCTACGACACCTACTA	Bases altered to introduce mutation underlined, this study
*aaaA*::H100Ar	GAACTTCCCGCTGCTGCCG	
*aaaA*::D102Af	GCGCGCACTACGCCACCTACTACGGTCGC	Bases altered to introduce mutation underlined, this study
*aaaA*::D102Ar	CGAGTACCAGGAACTTCCCGCTGCTGCCG	
*aaaA*::E147Af	GGCGCCGCCGAGGAAGG	Bases altered to introduce mutation underlined, this study
*aaaA*::E147Ar	GAAACCGACCACCTCGAGGCCGTT	
*aaaA*::E149Af	CGAGGAGGCCGGCCTGCG	Bases altered to introduce mutation underlined, this study
*aaaA*::E149Ar	GCGCCGAAACCGACCACCTCG	
*aaaA*::D176Af	ATGATCAACCTCGCCAGCCTGGTCACC	Bases altered to introduce mutation underlined, this study
*aaaA*::D176Ar	TCCCAGCAGGTTGGCGCGCT	
*aaaA*::H277AfB	TCAACCTGGGCCGACC	Bases altered to introduce mutation underlined, this study
*aaaA*::H277ArB	GCCGCCGGGAATC	

Primers are shown with the 5′ terminus on the left.

In the first instance, a phenotypic comparison between PAO1 and the derived PAO1Δ*aaaA* mutant was conducted to ascertain whether there were any gross changes in proteolytic capability. This analysis revealed that both PAO1 and PAO1Δ*aaaA* generated similar zones of clearing on agar plates containing skimmed milk, which correlated with no difference in casein degradation using the azocasein degradation assay (materials and methods, data not shown). Similarly there was no change in elastin degradation using the elastin-congo red degradation assay or haemolysis on blood agar plates (materials and methods, data not shown). Having been unable to detect a difference in the degradation of proteins known to be broken down by *P. aeruginosa*, we sought to identify the specificity of the predicted aminopeptidase activity of AaaA. To do this, an assay used for a similar purpose with other members of the M28 family was employed. For this assay, amino acids linked to *p*-nitroanilide are incubated with the suspected peptidase. Aminopeptidase activity is detected when the pseudo-peptide bond of the *p*-nitroanilide derivative is broken, and the chromophore released (4-nitroanilide) is monitored by a change at 405 nm over time.

**Figure 4:**
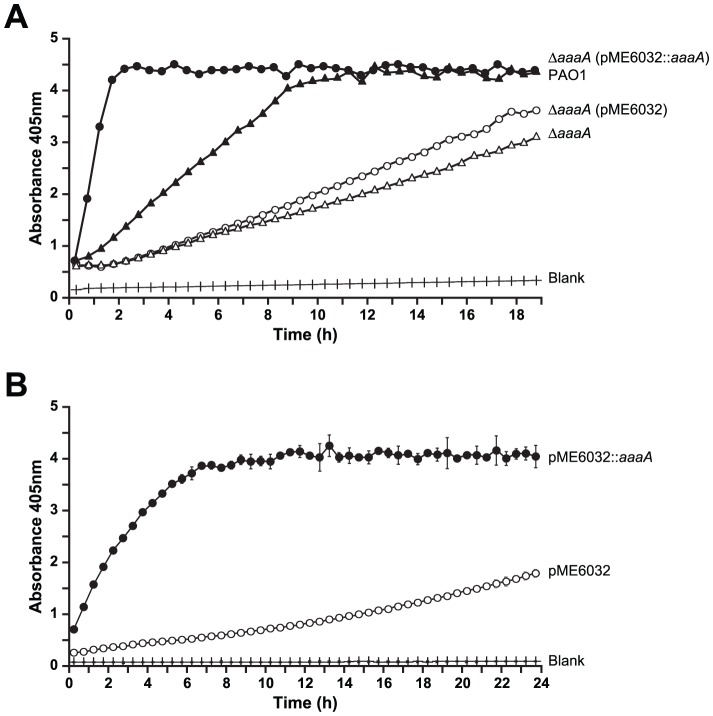
AaaA can remove arginine from *p*-nitroanilide. **Panel A.** The *P. aeruginosa* Δ*aaaA* mutant alone (open triangles) or bearing either the empty plasmid pME6032 (open circles) or its derivative carrying *aaaA* (pME6032::*aaaA*: closed circles) were treated as described in Figure S2B except arginine-*p*-nitroanilide was used as a substrate. WT PAO1 cells were treated similarly (closed triangles), and activities (measured as changes in A_405 nm_) are compared against a growth media blank (crosses). **Panel B.** *E. coli* DH5α bearing either the empty plasmid pME6032 (open circles) or its derivative carrying *aaaA* (pME6032::*aaaA*: closed circles) were grown in LB until exponential phase, induced with 1 mM IPTG, and then incubated with arginine-*p*-nitroanilide as described in Figure S2B. Activities are compared against a growth media blank (crosses). Error bars are+/−1 S.D. (n = 15). All measurements have been corrected for differential growth of bacteria by normalising to an initial OD_600 nm_ of 0.1.

Both PAO1 and PAO1Δ*aaaA* were only able to degrade methionine-*p*-nitroanilide and leucine-*p*-nitroanilide slowly and to a limited extent during 24 h, whilst a commercial preparation of SGAP clearly released maximal levels of 4-nitroanilide from leucine-*p*-nitroanilide and methionine-*p*-nitroanilide in less than 2 h (Figure S2 A,B,C). As one of the closest homologues (57% sequence identity in the passenger domain) of AaaA, the IAP aminopeptidase produced by *E. coli*, enables isoenzyme conversion of alkaline phosphatase by removing the terminal arginine residue from each protomer within the alkaline phosphatase dimer [50] and because *aaaA* expression levels are altered in the absence of the arginine-dependent regulator ArgR [51], we hypothesized that AaaA may release arginine from peptides. To test this, we assayed the ability of PAO1 and PAO1Δ*aaaA* to release 4-nitroanilide from arginine-*p*-nitroanilide. Whilst the wild type efficiently degraded the arginine-*p*-nitroanilide to fully release 4-nitroanilide within 8 h, the PAO1Δ*aaaA* mutant did not do this, even after 24 h of incubation (Figure 4A). To verify that this difference was due to the deletion of *aaaA*, complementation was performed with plasmid pME6032::*aaaA*. In comparison to the empty vector (pME6032), pME6032::*aaaA* significantly repaired the mutant's ability to degrade arginine-*p*-nitroanilide, giving higher levels of degradation than the wild type.

To confirm that the arginine-*p*-nitroanilide degradation was a direct consequence of AaaA, the activity of AaaA overproduced in the heterologous host *E. coli* was measured. As can be seen in Figure 4B, maximal release of 4-nitroanilide was achieved within 5 h by *E. coli* (pME6032::*aaaA*) whilst *E. coli* (vector) did not degrade arginine-*p*-nitroanilide significantly within 24 h. Together these data strongly suggest that AaaA is an arginine-specific aminopeptidase.

### Site directed mutagenesis reveals key residues in AaaA are involved in aminopeptidase activity

To establish that the predicted active site within AaaA is responsible for the measured arginine-specific aminopeptidase activity, site directed mutagenesis targeted at predicted key residues was undertaken. To identify key residues in the catalytic site, we took advantage of the crystal structure of SGAP (referred to as 1xjo on the protein structure database). Alignments (Figure 3A) had shown that residues involved in catalysis were conserved between AaaA and SGAP, so these were mapped onto the crystal structure (Figure 3B). It was evident from this that the active site pocket was well conserved, as were key residues stabilising structural elements.

Seven residues were chosen (underlined in Figure 3A), and site directed mutagenesis used to convert them to alanines. Six of the residues mapped within the predicted catalytic pocket of AaaA (H100, D102, E147, E149, D176, D277), and the other was located on a surface exposed, unstructured loop (G89) (Figure 3C). Three of the mutants with amino acid substitutions predicted to lie within the active site pocket (H100, E147, E149) were overproduced in *E. coli* as stable proteins of the predicted size (Figure 3D). Two of these were localised to the outer membrane (E147 and E149, Figure 2). All the mutant proteins, except G89A (the only residue not predicted to be in the active site) exhibited a reduced ability to degrade arginine-*p*-nitroanilide (Figure 3D) confirming that catalysis occurred in the predicted active site pocket.

### The *ΔaaaA* mutant is unable to grow when the sole source of carbon and nitrogen is provided by peptides with amino terminal arginine

**Figure 5:**
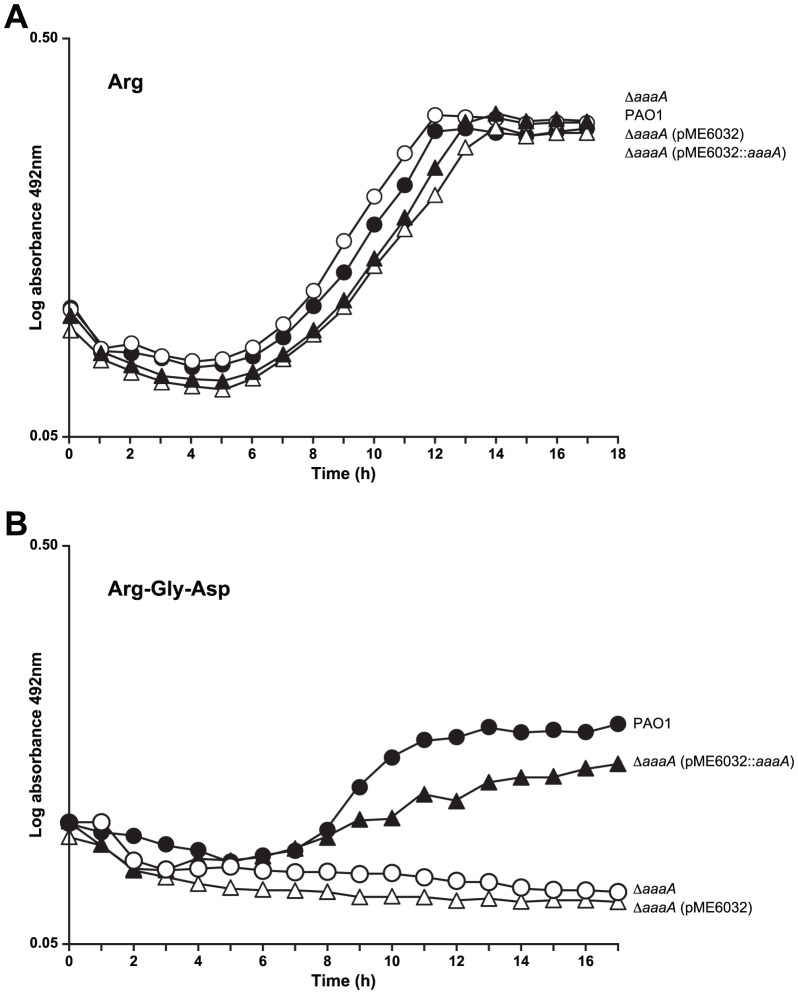
The activity of AaaA enables *P. aeruginosa* to grow using the tripeptide arg-gly-asp as the sole source of carbon and nitrogen. *P. aeruginosa* PAO1 (closed circles) and its derived *aaaA* deficient mutant (Δ*aaaA*, open circles) alone or bearing pME6032 (vector, open triangles) or pME6032::*aaaA* (complemented, closed triangles) were grown to mid-exponential phase before the induction of AaaA production by 1 mM IPTG. Cells were resuspended in MMP to OD_600_ of 1, and subsequently 20 µl of this solution diluted into 200 µl of MMP containing arginine at 10 mM (**Panel A**), or 10 mM of the tripeptide arg-gly-asp (**Panel B**). The graph shows the subsequent growth in the Tecan monitored by observing the increase in OD_492_ over time. The data is representative of 3 independent repetitions of this experiment.

*P. aeruginosa* is able to use arginine as the sole source of carbon and nitrogen. We therefore thought it possible that by releasing arginine from peptides, AaaA could provide a valuable source of arginine to be fed into metabolism for growth in specific environmental conditions. In rich media sources of carbon and nitrogen are plentiful. It was therefore no surprise that the Δ*aaaA* mutant grew similarly to its parent in rich media (Figure S1B). To establish whether the Δ*aaaA* mutant could import and metabolise arginine as well as its parent, we compared the growth of both in minimal medium (MMP) containing arginine as the sole carbon and nitrogen source [52], [53]. As can be seen in Figure 5A, both PAO1 and PAO1Δ*aaaA* grew equally well.

Interestingly, and in support of our hypothesis, when the peptide Arg-Gly-Asp was included in MMP as the sole source of carbon and nitrogen, the Δ*aaaA* mutant could not grow although the wild type *P. aeruginosa* PAO1 grew well (Figure 5B). The presence of the complementation vector (pME6032::*aaaA*) restored the growth of the Δ*aaaA* mutant to a level close to wild type (Figure 5B), indicating that there were no second site mutations underlying the growth deficiency observed in the Δ*aaaA* mutant.

**Figure 6:**
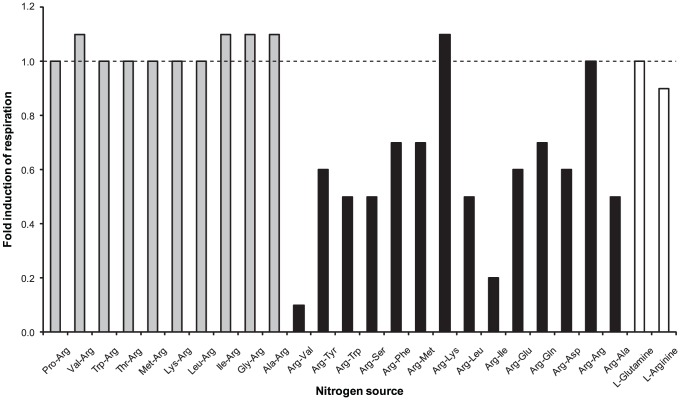
AaaA promotes the ability of *P. aeruginosa* to respire dipeptides with N-terminal arginine except when adjacent to Arginine or Lysine. *P. aeruginosa* PAO1 and its derived *aaaA* deficient mutant were inoculated into nitrogen minimal media (NMM) alone or NMM containing the indicated nitrogen source. Cellular respiration/metabolic activity is reported via reduction of tetrazolium dye and plotted against time. The area under the curve (AUC) for a selection of nitrogen sources following 24 h incubation in each condition is plotted here. The values have been normalised by subtraction of the AUC of the control (no nitrogen source added) on the respective Biolog plate. Relative respiration is calculated by the difference between the normalised AUC of wild type and mutant divided by their sum and multiplied by 100. The fold induction was calculated by dividing the normalised AUC of the mutant by that of the wild type, so a value of 1.0 is no change. Biolog Phenotype microarray plates PM03B and PM06-08 were used as indicated, and each condition performed in duplicate (results from one are shown).

Taking advantage of the growth deficiency exhibited by the Δ*aaaA* mutant, we were able to further define the specificity of the AaaA peptidase. The Phenotype Microarrays (Biolog Inc) enable the simultaneous comparison of a range of two thousand phenotypes including substrate utilisation and various chemical sensitivities, using cellular respiration as a reporter. We screened the Δ*aaaA* mutant and its parent, PAO1, to compare their ability to utilise 380 nitrogen sources including 24 arginine-containing dipeptides. The data is plotted as respiration over time (data not shown), and the area under these curves (AUC) has been calculated and normalised by subtraction of respiration in the absence of a nitrogen source in order to enable determination of fold induction of respiration in the mutant (Figure 6). Both PAO1 and PAO1Δ*aaaA* respired poorly in the absence of a nitrogen source and mirrored each other on a number of other nitrogen sources. For example, both PAO1 and PAO1Δ*aaaA* respired similarly if L-arginine was provided, whilst in the presence of lysine respiration was on a par with the negative control in which no nitrogen source was provided (Figure 6, data not shown). For all the non dipeptide nitrogen sources, if respiration levels indicated utilization (AUC>negative control), PAO1 and PAO1Δ*aaaA* respired equally well (fold induction ∼1), or the mutant respired better (fold induction >1). Notably, the wild type and Δ*aaaA* mutant respired equally well with all the dipeptides where arginine was placed at the *C*-terminus (fold induction ∼1). In contrast, with the exception of Arg-Arg and Arg-Lys dipeptides as the source of nitrogen, all the dipeptides with an amino terminal arginine supported better respiration of PAO1 than of the Δ*aaaA* mutant (Figure 6). The extent of this phenotypic difference observed between the wild type and Δ*aaaA* mutant varied slightly depending on the dipeptide. The greatest difference in the respiration of the Δ*aaaA* mutant compared to the WT was exhibited with the dipeptides containing Arg-Ile and Arg-Val (Figure 6)

### The Δ*aaaA* mutant is deficient in long term survival in a mouse chronic wound infection model

**Figure 7:**
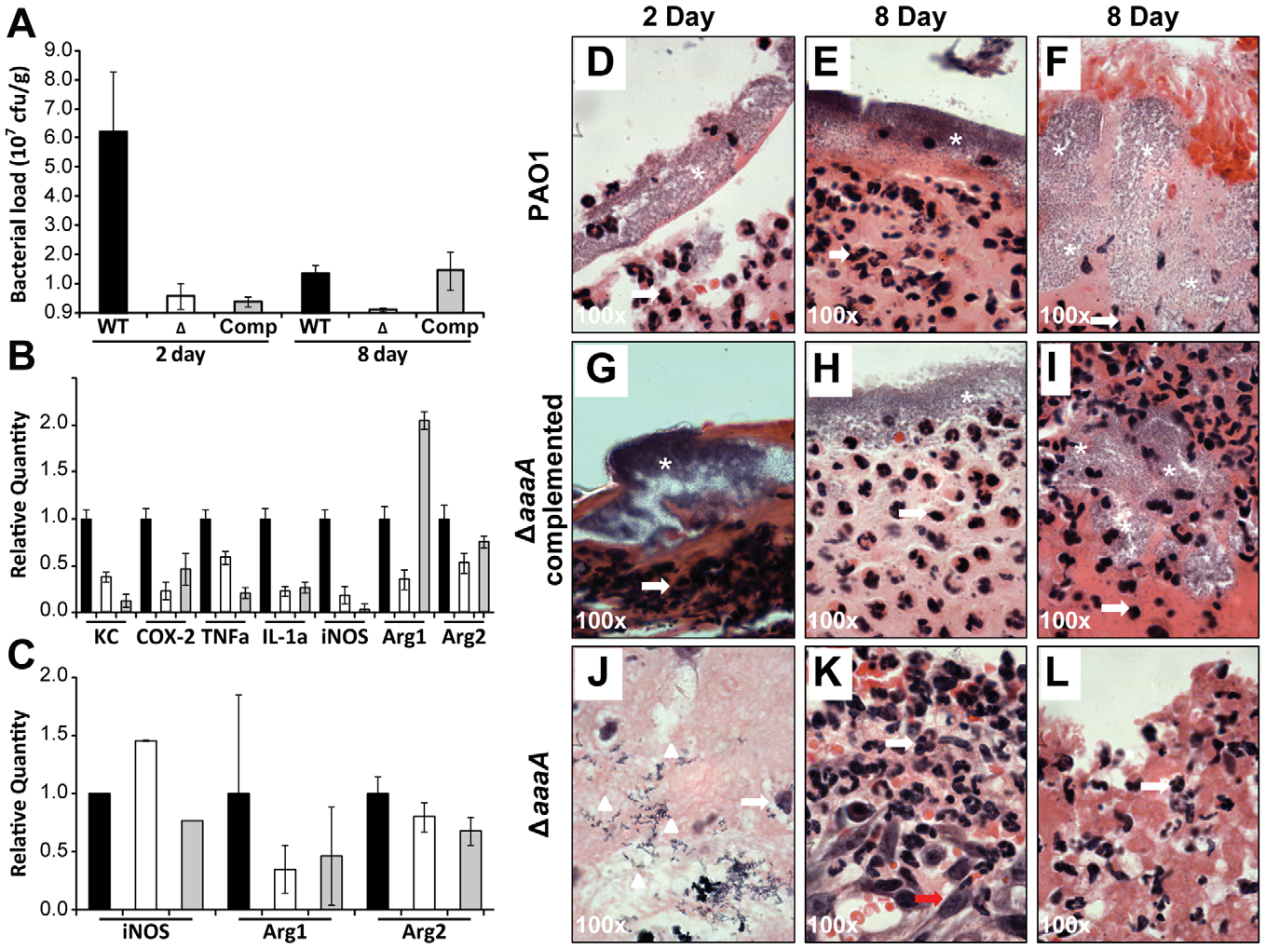
The AaaA deficient mutant is less virulent in the chronic mouse wound model. Either the *P. aeruginosa* wild type PAO1 (black bars), the Δ*aaaA* mutant (white bars), or the complemented Δ*aaaA* mutant PAJL2 (grey bars) was inoculated (10^4^ CFU) into a chronic wound in each of 9 mice. After 2 (3 mice per group) or 8 (7 mice per group) days, wound tissue was removed and the bacterial load was estimated by calculating the colony forming units (**Panel A**). Chronically-wounded mice were euthanized at post infection day 2 (3 mice per group) or day 8 (7 mice per group), and wound tissue was harvested for qRT-PCR to detect the mRNA of the indicated cytokines and other host enzymes in the infected wound tissue as described in the materials and methods (**Panel B and C**). Tissue from the wounds of the 2 day infected mice (**Panels D,G,J**) or 8 day infected mice (**Panels E,F,H,I,K,L**) was stained with H&E and is shown at 100× magnification. Images of the *P. aeruginosa* wild type PAO1 (**Panels D,E,F**), Δ*aaaA* mutant (**Panels G,H,I**), and the complemented Δ*aaaA* mutant PAJL2 (**Panels J,K,L**) are shown with infiltrating neutrophils indicated by white arrows, elongated fibroblasts with a red arrow, single bacterial cells with white arrow heads and clumps of bacteria with a white asterisk. **Panels D–E,G–H,J–K** are representative of the wound site and **Panels F,I,L** are representative of the site of infection below the wound.

Since ATs are notorious for their role in pathogenicity, we set out to establish whether AaaA contributed to *P. aeruginosa* virulence in an animal infection model. Although the wild type and Δ*aaaA* mutant were able to establish an acute infection in a mouse burn wound model equally well, there was a difference in virulence in a chronic mouse wound model [54]. As can be seen in Figure 7A, the Δ*aaaA* mutant showed significantly reduced survival in a mouse wound chronic infection in comparison to wild type both 2 days and 8 days post infection.

To discount the possibility of secondary site mutations attenuating virulence, *aaaA* and its adjacent promoter were introduced onto the chromosome of *P. aeruginosa* Δ*aaaA* using miniCTX1 to create a stable chromosomal complementation for the 8 day animal infection model (*P. aeruginosa* PAJL2). *In vitro*, PAJL2 grew similarly to PAO1 and the Δ*aaaA* mutant in LB (Figure S2), and degraded comparable levels of arginine-*p*-nitroanilide. Although behaving like the *ΔaaaA* mutant initially in the chronic mouse wound infection model, and exhibiting lower viable cell numbers (colony forming units, cfus) than the WT at 2 days post infection, by 8 days post infection the number of viable bacteria isolated from the wound site for PAJL2 more closely resembled the WT than the Δ*aaaA* mutant.

In addition to an alteration of bacterial load within a mouse wound chronic infection, there were also changes in immune response dependent on the presence of AaaA. Initial analysis revealed that lower levels of expression of the pro-inflammatory cytokines TNFα and IL-1α were found in mice colonised with the Δ*aaaA* mutant in comparison to the wild type by RT-PCR (data not shown). More sensitive qRT-PCR confirmed this, and in addition showed that the expression of KC and COX-2 were also lower in mice colonised with the Δ*aaaA* mutant in comparison to the wild type 2 days post infection (Figure 7B).

To investigate the expression of more host factors including components of the innate immune system linked to arginine availability in the tissue, the expression of iNOS, Arg1 and Arg2 was quantified and also found to be lower within the mouse wound infected by the Δ*aaaA* mutant in comparison to the wild type 2 days post infection (Figure 7B). In each case host factor expression in skin infected by the complemented Δ*aaaA* mutant matched that infected by the Δ*aaaA* mutant rather than the WT which is in line with the cfus with the exception of Arg1, which exhibited elevated expression in the complemented Δ*aaaA* mutant 2 days post infection.

Levels for the cytokine expression at day 8 of the infection are not shown as levels are low at this point in the infection (data not shown). As a first step to understanding the potential mechanism underlying AaaA function in the context of chronic skin infections, the expression levels of iNOS and Arginase are shown. During trauma, such as in a skin wound, mammalian arginine requirements exceed production [55]. Thus, with limited arginine available, release of arginine from peptides by AaaA may disrupt a delicate balance of arginine utilization in host cells. Arginase and iNOS use arginine as a common substrate, and compete with each other for this substrate [56]. Although a much more extensive investigation is required to obtain statistically significant data of expression collected in parallel to protein levels and activity, there was an interesting trend. After 8 days of infection, the levels of iNOS expression rose in mouse skin infected by the Δ*aaaA* mutant compared to the WT and complemented mutant whilst expression of Arg1 tended towards a fall in the Δ*aaaA* mutant. The error in the measurement of Arg1 at 8 days post infection is higher than for the other genes analysed, thus data must be viewed with caution although loose trends can be identified to inform further studies and maximise the benefit of using an animal model. Whilst iNOS expression in the skin infected by the complemented Δ*aaaA* mutant mirrored that in PAO1 8 days post infection, Arg1 expression did not. However, interestingly, the Arg1 induction evident 2 days post infection had resided by 8 days. Arg2 expression does not appear strongly influenced by the course of infection, perhaps due to its different tissue distribution or cellular localisation [56].

Sectioning the colonised skin and staining with hematoxylin and eosin (H & E) indicated that neutrophils infiltrated all the infected skin wounds (Figure 7, Panels D–L). Consistent with the lower cfus, it was difficult to visualise the bacteria in the Δ*aaaA* mutant infected skin (Panels J–L), and they could not be located in the skin 8 day post infection. Interestingly, clumps of bacteria could be found within the mouse skin sections for PAO1 and the complemented ΔaaaA mutant after 2 or 8 days (see asterisk in Panels D–I). In contrast, bacteria observed for the Δ*aaaA* mutant infection after 2 days, were not located within dense clumps. Moreover, there was evidence of wound repair for the skin infected by the Δ*aaaA* mutant after 8 days as infiltration by fibroblasts was observed (Panel K).

## Discussion

Here, we show that *P. aeruginosa* possesses an aminopeptidase, AaaA. AaaA is an AT that is tethered to the surface of *P. aeruginosa* and specifically removes amino terminal arginine from peptides. Site directed mutagenesis revealed that AaaA aminopeptidase function relies on key amino acids that are conserved within the M28.005 aminopeptidase family and are located within the active site pocket of SGAP [40], [43]. Although no specific target peptides or proteins could be identified, we showed that AaaA released arginine from the aminoterminus of di and tripeptides. *P. aeruginosa* was able to use the liberated arginine as a nutrient for growth, providing a fitness advantage when arginine-containing peptides were the sole source of nitrogen in the environment. Colonisation experiments in mice revealed that whilst AaaA did not confer a virulence advantage in an acute burn wound infection, it did in a chronic wound infection. The observed attenuation of the Δ*aaaA* mutant was associated with reduced levels of the cytokine expression.

The M28 family includes amino and carboxy-specific peptidases with a range of different specificities that are produced by a diverse array of organisms (http://merops.sanger.ac.uk). These include eukaryotes (e.g. humans, mice, plants and nematodes), as well as bacterial species, including but not limited to *Streptomyces*, *Escherichia*, *Vibrio*, and *Pseudomonas*. Despite this diversity of hosts, the residues of the active sites are conserved [39], [49], [57], [58], suggesting a common reaction mechanism. The crystal structure and catalytic mechanism of one of the M28 aminopeptidases, SGAP, has been elucidated [43]. The residues that maintain the correct active site conformation were identified, and it was proposed that a glutamic acid residue plus either a tyrosine or histidine brought about the formation of the catalytic complex via interaction with two zinc ligands [40], [59]. Our data suggests that the active site of AaaA incorporates the conserved active site residues of the M28 family. Moreover, two of them (E147 and E149) appear to have roles in catalysis or stabilization of the active site pocket since mutation of them to alanine rendered AaaA stable, OM localised, but non-functional. Presumably the mutant proteins are unable to form the enzyme-substrate intermediary complex. We cannot currently conclude whether AaaA utilizes a similar or distinct catalytic mechanism to SGAP since the predicted equivalent of one of the two SGAP catalytically important active-site residues (Y246) has yet to be mutated in AaaA. Moreover, the equivalent of the other SGAP active site residue (E131) in AaaA (E147) generated a mutant protein that retained 46% activity when replaced by alanine [40]. From the alignment in Figure 3A however it can be seen that E147 is adjacent to two other glutamates, whilst SGAP has a run of only two. It is possible that one the neighbouring glutamates may be able to substitute as a general base in AaaA in the E147A mutant. Since E149A retained only 7% activity, it may plays a dominant role within the active site. However, it is not yet possible to deduct whether this is for co-ordination of the zinc ions or in catalysis. Since the H100A mutant was not localised correctly to the OM, its loss of activity (down to1.5%) could be attributed to mislocalisation (Figure 2).

The instability of a subset of the active site mutants cannot currently be explained. Point mutations in ATs produced by other bacteria have not led to degraded proteins, so this was unexpected and interesting. Studies are underway to confirm the localisation of these degraded mutant proteins and we aim to determine the nature of the stable products observed. Moreover, we predicted that the C-terminal autotransporter β-barrel domain of AaaA would have been protected from digestion from exogenous trypsin due to membrane embedding, and thus parallel studies will investigate why attempts to detect it have not yet been successful and why AaaA migrates at a molecular mass approximately 10 kDa larger than predicted. It is not surprising that the passenger domain could not be detected after trypsin digestion in the supernatant as the software PeptideCutter (http://web.expasy.org/peptide_cutter/) indicated that 27 residues can be digested by trypsin. These were mapped onto the 3D model of PA0328 using RasMol software, where it was possible locate 14 of these 27 residues on the surface, thereby indicating that they are more accessible to digestion by trypsin (residues: 33, 38, 47, 106, 130, 136, 185, 262, 273, 277, 279, 283, 309 and 329). The biggest fragments of PA0328 which could be released by such digestion would have a predicted molecular weight of 8.3, 6.2 and 5.6 kDa, which are too small to be detected.

The mouse wound infection model has reliably identified virulence factors by revealing attenuated mutants [54], [60]–[72]. The altered pathogenicity in the chronic wound model was associated with lower bacterial loads and levels of the proinflamatory cytokines IL-1α, KC, TNFα, and COX-2. The reduction of TNFα with a lower level of colonisation is in line with a previous report suggesting that TNFα is up-regulated during chronic *P. aeruginosa* infection [73]. The importance of IL-1 in the defence against *P. aeruginosa* is supported by the reduced survival of IL-1-deficient mice following colonisation with *P. aeruginosa* [74], supporting the hypothesis that the presence of IL-1 and TNFα is disadvantageous to the survival of *P. aeruginosa* and thus selection for the acquisition of secreted proteases that actively degrade these cytokines [75]. Both KC and COX-2 have also been linked with the progression of *P. aeruginosa* infection [76], [77], and microbial load may be influencing the expression of all these cytokines. The influence of microbial load on cytokine levels is in part indicated by the data obtained from the complemented *ΔaaaA* mutant. At day two post infection, the number of viable cells for the *ΔaaaA* mutant and complemented *ΔaaaA* mutant were similar, as were all the cytokines, however by day 8 post-infection the complemented *ΔaaaA* mutant resembled PAO1 in viable cell numbers, although cytokine levels were not determined. It is not clear why the behaviour of the complemented *ΔaaaA* mutant did not mirror that of PAO1 exactly, but it could be due to the ectopic localisation of *aaaA* and its promoter since the local chromosomal structure or features may influence expression of *aaaA*.

**Figure 8:**
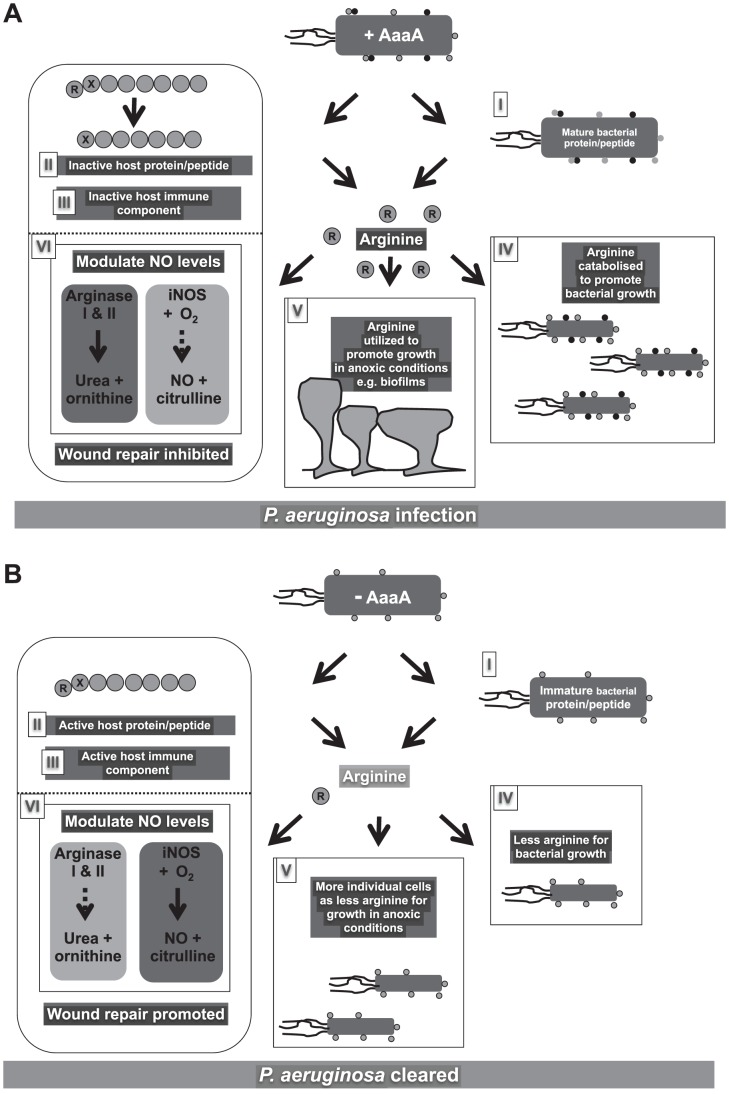
Cartoon Model illustrating a selection of the potential roles AaaA may have within a chronic wound. In **Panel A**, the *P. aeruginosa* WT scenario is depicted, where AaaA (black dots) is present on the surface of *P. aeruginosa* cells colonising a host. **Panel B** shows infection with an AaaA deficient mutant that only has non-AaaA proteins on its surface (grey dots). It is possible that AaaA may: **Panel I** degrade a protein on the surface of *P. aeruginosa*, causing activation that aids infection (represented by removal of the black outline around the grey dots in Panel A, but not in Panel B), **Panel II** degrade a host protein/peptide, that may be a component of the host immune system (**Panel III**) by removing an aminoterminal arginine (R in circle). These activities may be sufficient to aid pathogenicity, however they may serve to liberate arginine that can be catabolised by the bacteria (**Panel IV**) resulting in growth promotion in Panel A that is not evident in the absence of AaaA (Panel B). This may provide a fitness advantage to the bacteria that improves virulence. In conditions where oxygen is limited, the arginine may provide a particular advantage (**Panel V**), potentially enabling formation of biofilms that could both serve to promote colonisation and provide resistance against the immune system. If only some of the released arginine is utilized by the bacteria, local arginine levels may rise in the host (**Panel VI**). This could induce arginase production in host cells (depicted by dark grey box and solid black arrows in Panel A:VI). The arginase enzymes will degrade the arginine, reducing its availability as a substrate for iNOS (indicated by pale grey box and dashed grey arrows in Panel A:VI). Consequently, there will be lower levels of nitric oxide (NO) and *P. aeruginosa* will be able to successfully establish an infection. Alternatively, in Panel B:VI, AaaA is absent from the invading *P. aeruginosa*, so there is no degradation of proteins and peptides. This maintains the limited arginine concentration and avoids induction of arginase in host cells. Consequently, arginine would be available to serve as a substrate for iNOS, and the nitric oxide generated could disable the bacterial cells and promote wound healing.

There are a number of potential underlying mechanisms that could lead to reduced pathogenicity of the Δ*aaaA* mutant, and these are depicted in the cartoons in Figure 8. Firstly, the lack of AaaA may lead to reduced fitness *in vivo*. The data presented here clearly shows that AaaA enables *P. aeruginosa* to release arginine from the aminoterminus of peptides and feed this into metabolism to enable growth *in vitro*, and could thus provide the strength of numbers to overcome the host defences.

The fitness advantage extended by liberating arginine could be of particular relevance in anaerobic conditions since in the absence of oxygen, nitrate and nitrite, *P. aeruginosa* is able to catabolize arginine by substrate level phosphorylation to serve as an energy source for anaerobic growth [78], [79]. The presence of arginine in the environment triggers the regulator ArgR to respond by activating a set of genes involved in arginine metabolism or uptake [80]. ArgR works in concert with the anaerobic regulator (ANR; [53]), and interestingly has been shown to positively regulate *aaaA* 3.7 fold [51]. In this way arginine metabolism and growth in anaerobic environments are linked with each other [81] and AaaA.

Additional information linking AaaA to anaerobic growth can be inferred by considering the lifestyles of bacteria which harbour AaaA homologues. For example the uncharacterised *Pectobacterium carotovorum* AT ECA2163 is a member of the M28.005 sub-family with 64% sequence identity with AaaA. *P. carotovorum* is well known for its ability to grow in anaerobic environments [82]. Another observation that could argue for a link between AaaA and anaerobic growth is the absence of a homologue of AaaA in the plant pathogen *P. syringae* DC3000. This is despite the presence of 9 ATs in *P. syringae* [83], two of which are homologues of the other AT proteins in *P. aeruginosa*. Notably, *P. syringae* DC3000 is known for its virulence in aerobic conditions within the apoplast of tomato (an environment with particularly low levels of arginine). *P. aeruginosa* colonises many sites where anaerobic or microaerobic conditions are encountered, both in the environment e.g. water-logged soils and sediments and *in vivo* e.g. wound infections, otitis media, and pulmonary infections including those seen in CF patients [62], [84]–[87]. [88].

Although the Δ*aaaA* mutant was not attenuated in the acute mouse burn wound model, there was a significant attenuation in the chronic wound model. The latter, probably more so than the burn model [54], relies on the formation of biofilms that enable persistence and immune cell evasion [89], [90]. Indeed, dense clusters of bacteria which may represent biofilms were observed in mouse skin infected by the wild type and complemented Δ*aaaA* mutant, but were absent from wounds infected by the Δ*aaaA* mutant. A possible explanation could be that there is a growth advantage conferred by AaaA under anaerobic stress or within biofilms which leads to increased resistance to attack by immune cells. We are currently investigating the biofilm formation of the Δ*aaaA* mutant *in vitro* and *in vivo* to determine whether this could be a factor.

There is already a precedent for a pathogenicity mechanism involving a fitness advantage provided by a protease generating arginine in anaerobic conditions: the Gram-negative bacterium *Porphyromonas gingivalis* relies on the production of an arginine-specific gingipain peptidase to survive in the anaerobic pockets within gums where it causes chronic inflammation and destruction of the bone in which teeth are embedded. Since *Por. gingivalis* cannot utilise sugars, it has an absolute requirement for proteins as a carbon and nitrogen source. The degradative gingipains, are thus an absolute requirement for growth on medium containing proteins such as bovine serum albumin [91]. In addition to endopeptidase activity, the arginine gingipain has an arginine-specific aminopeptidase activity akin to that of AaaA [92], and we have shown that AaaA is required for growth in certain environments.

Alternatively the loss of AaaA may influence virulence because one of its functions is to activate a bacterial virulence factor in a similar way to which the AT NalP activates other ATs in *Neisseria meningitidis* [93]. It is also possible that AaaA may utilise its aminopeptidase activity to inactivate a host protein and thereby aid pathogenicity of *P. aeruginosa*. Such a mechanism would be analogous to the modification of the extracellular matrix by the AT Hap which aids attachment of *Haemophilus influenzae* [94]. Another way that AaaA might contribute to virulence of *P. aeruginosa* is by inactivating or degrading immune system components. This would be akin to one of the roles of the *Por. gingivalis* gingipains which are involved in direct degradation of host immune factors including cytokines, immunoglobulins and complement factors [95]–[97]. Further study is required to assess whether the lack of any of these mechanisms underlie the attenuation of the Δ*aaaA* mutant in the chronic mouse model.

Recently, modulation of the arginases as a means to enable microbial pathogenesis has been put forward [56], and suggests an additional interesting hypothesis to explain why AaaA plays such a key role in pathogenicity of *P. aeruginosa*. The limited arginine availability within skin wounds [55] creates a delicate balance of arginine utilization within host cells exploited by a range of pathogens [56]. Arginine can be used as a substrate by host cell Arginase I and II enzymes which differ in their tissue distribution and subcellular localization [56]. Both arginase isoforms release urea and ornithine for metabolism and this leads to the T_H_2 alternative activation if it occurs in macrophages. Arginine is also a substrate for the inducible nitric oxide synthase (iNOS). The iNOS enzyme combines arginine and oxygen to form nitric oxide which inhibits bacterial growth forming part of the T_H_1 classical pathway of macrophage activation. Elevated levels of arginine stimulate arginase activity in the host and bacterial cells [98]. This will favour the breakdown of arginine to urea and ornithine. This will, in turn, reduce the inducible nitric oxide synthase (iNOS) response because there will no longer be arginine available to act as a substrate for iNOS [99]. In addition to lowering the amount of nitric oxide to fight the pathogen, since nitric oxide aids wound healing [55], its absence will prevent the host regenerating physical barriers to hinder bacterial colonisation (Figure 8). Whilst a rigorous study involving infections of mice mutated in the arginase pathway and inhibitors of AaaA is required to establish this potential mechanism, the data presented provides tantalising preliminary data that supports this model. In Figure 7C Arg1 and iNOS expression were slightly reduced and increased respectively in the Δ*aaaA* mutant compared to the wild type, and in Figure 7K, an influx of fibroblasts is observed suggesting that wound healing is occurring.

In support of AaaA releasing arginine from peptides to tip the balance of host cells towards degrading it via arginase enzymes rather than producing NO using iNOS (see [100] for a comprehensive overview of the regulation of immune responses by L-arginine), mice deficient in iNOS and TNFα clear *P. aeruginosa* from their lungs less efficiently in a malnourished CF infection model [101]. Interestingly, another important pathogen (the stomach dwelling *Helicobacter pylori*) encodes a constitutive arginase (RocF) that functions in exactly the manner outlined above, although the RocF arginase is produced directly by the bacterium. In contrast, our model suggests that the altered arginine levels generated by AaaA stimulate expression of the host arginase. RocF consumes arginine and prevents NO production in cultured macrophages, which is relevant to pathogenicity because the *rocF* mutant is more efficiently killed and eliminated by activated macrophages [102]. Similarly, removal of arginine from the oral cavity through introduction of *Lactobacillus brevis* producing arginine deiminase led to reduced generation of nitric oxide which was associated with reduced inflammation and has been proposed as a novel therapeutic to combat periodontal disease [103]. In contrast to these examples, we are proposing that rather than acting as an arginase directly, AaaA is generating arginine that stimulates the expression of the host arginase (an approach taken by a range of intracellular pathogens [56]). For this to occur, the arginine released from peptides by AaaA on the surface *of P. aeruginosa* would need to remain extracellular rather than being directly imported into the bacterial cell and used in metabolism. It is possible that we observed enhanced bacterial growth/respiration due to the presence of AaaA *in vitro* by virtue of the growth conditions employed, which would be very different *in vivo*. Further investigation of this will require technology capable of monitoring metabolites at a single-cell resolution during an infection *in situ*. Linking this to iNOS and arginase expression would be ideal to provide an explanation for the delay in stimulation of iNOS in the Δ*aaaA* mutant compared to PAO1 since this was seen at 8 days, but not 2 days post infection in the mouse skin (Figure 7B,C).

Clearly AaaA is located on the surface of *P. aeruginosa*, probably by virtue of its AT domain, and has arginine-specific aminopeptidase activity that can be used to release arginine to provide a growth fitness advantage. Whilst we were able to show that loss of AaaA led to attenuation in a mouse chronic wound infection, there remains a number of interesting hypotheses that might explain the underlying mechanisms involved in this role in pathogenicity.

Further understanding of the reaction mechanism is also critical as aminopeptidases play central roles in several disease states (e.g. stroke, diabetes, cancer, HIV, neuropsychiatric disorders) and other bacterial infections. Since several naturally occurring hydroxyethyl isostere dipeptide metallo-aminopeptidase inhibitors (e.g. bestatin, leuhistin and actinonin) alleviate disease symptoms, e.g by inhibiting matrix degradation and invasion of extracellular matrixes by fibrosarcoma cells or decreasing HIV viral load, metallopeptidases are ideal targets in the search for novel therapeutic drugs. Furthermore, these enzymes have important biotechnological applications in the processing of proteins and can be exploited as a diagnostic tool [45], [46].

## Materials and Methods

### Ethics statement

This study was carried out in strict accordance with the recommendations in the Guide for the Care and Use of Laboratory Animals of the National Institutes of Health. The protocol was approved by the Institutional Animal Care and Use Committee of Texas Tech University Health Sciences Center (Protocol Number: 07044).

### Strains and growth conditions

Bacterial strains (see Table 1) were routinely cultured in Luria Bertani (LB) Broth [104] at 37°C, shaking. Strains were routinely maintained on LB agar plates and kept frozen in glycerol (20% v/v) at −80°C. Growth curves were performed in 100 ml cultures shaken at 200 RPM in 500 ml conical flasks at 37°C, in microtitre plates monitored in a Infinite 200 (Tecan using Greiner 96 well flat black plates) or in Biolog phenoarrays (see below). Minimal Medium P (MMP) comprised Na_2_HPO_4_ 1.47 g, KH_2_PO_4_ 0.648 g, MgSO_4_ 0.2 g, FeSO_4_ 0.001 g per litre [52]. Antibiotics were used at the following concentrations: Ampicillin (100 µg/ml), Kanamycin (50 µg/ml), Chloramphenicol (37.5 µg/ml), and Tetracycline (20 µg/ml) for *E. coli* and Chloramphenicol (37.5 µg/ml), and Tetracycline (125 µg/ml) for *P. aeruginosa*. The tripeptide Arg-Gly-Asp (Sigma) was dissolved in MMP to a concentration of 20 mM and dispensed in 200 µl volumes to individual wells of a clear bottomed sterile 96 well plate (Costar). Bacteria from an overnight culture were resuspended to 1 OD_600_ unit and washed three times in MMP before inoculation into the MMP-tripeptide growth medium to 0.1 OD_600_. Cell growth was monitored at 37°C in an automated plate reader (Anthos Lucy 1), over a 24 h period. Arginine (10 mM) was utilized for growth using the same protocol described for the tripeptide above.

### Generation of AaaA specific antibodies

*E. coli* BL21[DE3](pET21a::*aaaA*) was grown to OD 0.5 and induced with 500 µM IPTG for 3 h. Cells were harvested by centrifuation at 6000×g for 10 min, resuspended in SDS-PAGE loading buffer (50 mM Tris-Cl pH 6.8, 100 mM DTT, 2% w/v SDS, 0.1% w/v bromophenol blue, 10% w/w glycerol) and separated through SDS PAGE, and stained with Coomassie blue (10% w/v Coomassie blue, 40% v/v methanol). The protein band of the expected molecular mass was electroeluted from the gel [105], verified as being AaaA by tryptic mass spectrometry (performed by the University of Nottingham Proteomics Service) and used to raise antisera by Harlan essentially as described previously [105]. Before using, the antisera was incubated with a cell lysate prepared from *P. aeruginosa* Δ*aaaA* mutant to absorb non-specific antibodies as previously described [105].

### Analysis of *P. aeruginosa* exoproduct production

The level of haemolysis induced was assessed by the size of the zone of clearing around colonies grown on Columbia blood agar (Oxoid). The level of milk protein proteolysis by LasB, alkaline protease and protease IV [106] was assessed by the zone of clearing around colonies grown on LB skimmed milk agar (LB agar+1% (w/v) skimmed milk). The production of elastase was monitored using elastin-congo red. This was performed by adding 100 µl of spent culture supernatant harvested from an LB overnight broth culture to 20–30 mg of elastin congo red (Sigma). Following addition of 1 ml 100 mM Tris-Cl/1 mM CaCl_2_ pH 7.5, incubation for 4 h at 37°C shaking, and removal of particulates by centrifugation at 13,000×g for 1 min, the optical density at 495 nm was measured.

### Nitrogen source utilisation by Phenotype Microarray

Nitrogen utilisation was analysed using the Phenotype MicroArray technology (Biolog inc). All fluids, reagents and PM Panels were supplied by Biolog and used according to the manufacturer's instructions. Briefly, bacteria were cultured for 16 h on Luria-Bertani agar plates at 28°C. Cells were harvested with a sterile cotton swab and suspended in 10 ml of inoculating fluid (IF-0), and the cell density was adjusted to 85% transmittance (T) on a Biolog turbidimeter. The minimal media inoculating fluid (IF-0a) contained 100 mM NaCl, 30 mM triethanolamine-HCl (pH 7.1), 2.0 mM NaH_2_PO_4_, 0.25 mM Na_2_SO_4_, 0.05 mM MgCl_2_, 1.0 mM KCl, 1.0 µM ferric chloride, and 0.01% tetrazolium violet [107]. Before the addition to PM microtiter plates, bacterial suspensions were further diluted into 12 ml of IF-0a (per plate) in the relevant inoculating fluid. The carbon source for PM03B, PM06-08 experiments that measure nitrogen and peptide utilization was 20 mM sodium succinate and 2 µM ferric citrate. Substrate utilization was measured via the reduction of a tetrazolium dye forming a purple formazan (supplied by Biolog) and is indicative of active cellular respiration at 28°C. Formazan formation was monitored at 15 min intervals for 30 h. Kinetic data were analyzed with OmniLog-PM software. Each experiment was performed at least twice per strain.

### Molecular manipulations

Small-scale preparation of plasmid DNA was performed with a plasmid purification kit (Qiagen). Chromosomal DNA was extracted from *P. aeruginosa* with Promega wizard genomic DNA kit according to the manufacturer's instructions. Restriction enzyme digestions, ligations (T4 DNA ligase, Promega), and agarose gel electrophoresis in 1× TAE buffer (80 mM Tris-acetate pH 7.8, 19 mM EDTA) were performed using standard methods [104]. Restriction fragments were routinely purified from agarose gels using the qiaquick kit (Qiagen). Transformation of *E. coli* was carried out by electroporation [108]. Conjugation into *P. aeruginosa* was performed by co-culturing *E. coli* S17-1λ*pir* donor bacteria with recipient bacteria on LB agar for 6 h at 37°C [109]. The oligonucleotide primers used in this study are listed in Table 1, and reactions were performed using gotaq DNA polymerase (Promega) with the following conditions unless otherwise stated. PCR cycles included a denaturation of 5 min at 96°C in initially and thereafter for 30 s followed by annealing for 30 s at a temperature adjusted according to the T_m_ of the primers and extension at the recommended temperature for the DNA polymerase for 1 min/1 kb amplicon. 30 cycles of amplification were employed followed by a 10 min final extension. Cloned PCR products were sequenced on both strands by Geneservice Limited (UK). Southern blotting was performed as described in [104] on genomic DNA digested for 3 h at 37°C. The probe was generated with the template pBluescriptΔ*aaaA* and primers aaaAfa and aaaArc. RT PCR was performed on RNA extracted from mouse tissue using Tri-Reagent (MRC, Cincinnati, OH) in accordance with the manufacturer's specifications. cDNA was prepared by combining 2 µg of total RNA, 400 U of SuperScript RT (Invitrogen, Carlsbad, CA), and 500 ng of oligo(dT)(Promega, Madison WI) and incubating the mixture at 42°C for 1 h, and boiling for 5 minutes at 95°C. Specific primer sets for genes encoding murine glyceraldehyde-3-phosphate dehydrogenase (GAPDH), cyclooxygenase-2 (COX-2), keratinocyte-derived cytokine (KC; an orthologue of IL-8), IL-1α and TNFα (Table 1) were used to amplify DNA templates in a TC-3000 thermocycler (Barloworld Scientific Staffordshire, United Kingdom) with *GoTaq* DNA polymerase (Promega, Madison WI). PCR products were run on 1.5% agarose gels containing 10,000 concentrate Gelstar (Lonza, Rockhand, ME), and the gels were visualized under UV light.

For quantitative real time PCR (qRT-PCR), RNA was extracted from tissues stored in RNALater (Ambion) using RNeasy Mini Kit (Qiagen) following standard procedures. RNA samples were treated with Turbo DNaseI (Ambion) and purified using the standard protocol of the RNeasy Minelute Cleanup Kit (Qiagen). RNA quality was assessed using the Bioanalyser 2100 (Agilent Technologies). RNA subsequently underwent cDNA synthesis using SuperScriptII (Invitrogen) and Random Primers (Invitrogen). cDNA was purified with MinElute PCR Purification Kit (Qiagen) following standard procedures. TaqMan Primer-Probes were selected from the Gene Expression Assays (Applied Biosystems) as follows Gapdh (Mm_99999915_g), Tnfα (Mm_00443258_m1), Il1α (Mm_00439620_m1), Arg2 (Mm_00477592_m1), Arg1 (Mm_00475988_m1), Ptgs2 (COX2) (Mm_00478372_m1), Clcx2 (KC) (Mm_04207460_m1), NOS2 (iNOS) (Mm_00440485_m1). All probes worked within 0.1 of the efficiency slope. Duplicate biological samples were used for each condition. PCRs were performed in triplicate 20 µl reactions using Gene Expression Master Mix (Applied Biosystems) and 10 ng of cDNA/well on an AB7500 (Applied Biosystems) all under standard procedures. PCR efficiencies were verified using standard curves.

### Bioinformatic analysis

Alignment of nucleotide and deduced amino acid sequences was performed using ClustalW2 (http://www.ebi.ac.uk/Tools/msa/clustalw2/). Homologous proteins were identified using BLAST tools (http://blast.ncbi.nlm.nih.gov/Blast.cgi) and peptidase family membership interrogated via the merops database (http://merops.sanger.ac.uk/). Alignments were submitted to the Swiss Model server to build 3D structures (http://swissmodel.expasy.org/SWISS-MODEL.html), or 3D structures were downloaded directly from the protein database bank (http://www.rcsb.org/pdb/home/home.dojsessionid=CA3F7454E9278A0456FCD6626F5BC692). 3D structures were viewed using RasMol Version 2.7.5.2 (Based on RasMol 2.6 by Roger Sayle Biomolecular Structures Group, Glaxo Wellcome Research & Development, Stevenage, Hertfordshire, UK), and using this software residues of choice were highlighted.

### Construction of plasmids used in this study

pBluescript::*aaaA* was constructed by PCR amplifying *aaaA* from the genome of *P. aeruginosa* PAO1 with primers aaaAstart and aaaAend and digesting them with *BamH*I and *EcoR*I to insert them into the same sites in pBluescript KS+. pDEST42::*aaaA* was created using the directions provided in the Invitrogen Gateway system. Essentially, the *aaaA* open reading frame was transferred from an Entry vector [110] into pDEST42 using the AttB1 sites. Insertions were screened by molecular weight following PCR amplification with AttB1-HIP and AttB2-HIP primers (Table 1), and verified by sequencing. To create the shuttle expression plasmid, pME6032::*aaaA*, primers aaaAstartEcoRI and aaaAendhis were used to amplify *aaaA* from pBluescript::*aaaA*. Following digestion with *EcoR*I and *Cla*I, the amplicon was inserted into similarly digested pME6032 (N.B one of the *Cla*I recognition sites of pME6032 was previously methylated to prevent cleavage). pET21a::*aaaA* was built by amplifying the *aaaA* open reading frame from pBluescript::*aaaA* with primers aaaAstartNdeI and aaaAend, digesting the amplicon and pET21a with *Nde*I and *Eco*RI, and ligating these together.

#### Generation of an in-frame deletion mutant of *aaaA*

Regions of DNA (approximately 600 bp long) immediately upstream and downstream of *aaaA* were amplified with primers aaaAfa/aaaArb and aaaAfb/aaaArc respectively. The amplicons were cloned into pBluescript KS+ using restriction enzymes *Xho*I/*Hind*III and *Hind*III/*Spe*I respectively (creating plasmids pBluescript::*aaaA*upstream and pBluescript::*aaaA*downstream respectively). The two DNA fragments were then excised with the same enzyme combinations, ligated together to generate an in-frame deletion at the *Hind*III site, between the *Xho*I and *Spe*I restriction enzyme recognition sites of pBluescript. The resultant plasmid, pBluescriptΔ*aaaA*, encodes only the first 5 and last 3 amino acids of the native AaaA separated by two residues (S and F), and was digested with *XhoI*/*SpeI* to excise the approximately sized 1.2 kb DNA fragment that was ligated into the suicide vector pDM4 [111] that had been similarly digested. The resultant plasmid (pDM4Δ*aaaA*) was electroporated into *E. coli* S17-1λ*pir* and chloramphenicol resistant colonies selected, which were conjugated to *P. aeruginosa* PAO1 (Nottingham). Transconjugant *P. aeruginosa* were selected by growth on *Pseudomonas* isolation agar (Difco) containing chloramphenicol. To select for the second crossover event, transconjugants were grown for 24 h at 37°C in LB broth containing 5% (w/v) sucrose. These cultures were subsequently streaked onto LB agar containing 5% (w/v) sucrose, and grown overnight at 37°C. Colonies which were obtained here were streaked onto LB agar plates containing chloramphenicol to ensure none retained Cm^R^ phenotype. The Δ*aaaA* mutant was confirmed by screening by PCR with primers aaaAfa and aaaArc which flank the region deleted. The absence of point mutations was verified by sequencing of this amplicon.

#### Generation of chromosomal complemented Δ*aaaA* mutant

*aaaA* and its upstream promoter were amplified using the primers aaaAminictxFor and aaaAminictxRev (see Table 1), and inserted into the pminiCTX1 multicloning site [112] following restriction digestion of the amplicon and vector with enzymes *NotI* and *EcoRV*. The resultant plasmid (pCTX::*aaaA*) was electroporated into the Δ*aaaA* mutant with selection on tetracycline, creating PAJL1. Flip recombinase-mediated excision of unwanted plasmid sequences was performed as described previously [112], creating the complemented *ΔaaaA mutant* (*ΔaaaA::CTXaaaA*) which was named PAJL2.

### Site directed mutagenesis

Desired site directed mutations were prepared using the Phusion site directed mutagenesis kit (according to the manufacturer's instructions, Finnzyme). The template used was pBluescript::*aaaA*. Following PCR with one mutagenic primer and one non-mutagenic primer (both 5′ phophorylated, see Table 1), ligation reactions with T4 DNA ligase (Promega) were performed for 3 h at 22°C. Products were electroporated into *E. coli* DH5α, and plasmids with the mutation (as determined by DNA sequencing), were amplified with primers aaaAstartNdeI and aaaAend, digested with *EcoRI*/*NdeI* to excise the mutated version of *aaaA* which was inserted into similarly digested pET21a (Novagen). In the case of the D102A mutation, direct subcloning was performed with *Bam*HI and *Eco*RI. For protein overproduction, the pET21a::*aaaA* derivatives were electroporated into *E. coli* BL21[DE3] or LEMO21, and induced at mid exponential phase with 1 mM IPTG (Sigma) for 1–3 h at 37°C with 200 RPM shaking.

### *p*-nitroanilide degradation assay

Stock solutions (20 mM) of *p*-nitroanilide derivatives (Sigma) were prepared. Arginine-*p*-nitroanilide hydrochloride was dissolved directly in MMP. Methionine*-p*-nitroanilide initially in ethanol:PBS (50∶50), whilst Leucine-*p*-nitroanilide and lysine*-p*-nitroanilide required initial solubilisation in methanol. All assays contained a final concentration of 1 mM of a *p*-nitroanilide derivative in MMP. Bacteria were resuspended to 1 OD_600_ unit and washed three times in MMP using centrifugation at 13,000×g for 1 minute and subsequent resuspension in a final volume of 1 ml of MMP. This suspension was used to inoculate 200 µl of MMP containing the *p*-nitroanilide reaction mixture in individual wells of sterile clear bottomed 96 well plates (Costar). Cells were inoculated to 0.1 OD_600_ and the degradation of *p*-nitroanilide monitored by observing OD_405_ for 24 h at 37°C in an automated plate reader (Anthos Lucy 1). If cell lysates were used as the source of enzyme, they were harvested into MMP, sonicated on ice for 10 seconds and the lysate was cleared by centrifugation at 13,000×g for 1 minute, with the resultant supernatant being added to the substrate and incubated as described above. In parallel, 0.027 units of active SGAP (Sigma catalogue number A9934) were incubated with the *p*-nitroanilides as substrates under the same reaction conditions.

### Trypsin treatment

Whole cells were collected by centrifugation for 5 min at 3,000×g, washed twice and resuspended to 1 OD_600_ units/ml in PBS-Hepes (0.1 M NaCl, 0.002 M KCl, 0.01 M Na_2_HPO_4_, 0.01 M KH_2_PO_4_ and 10 mM Hepes pH7.4) with or without Trypsin (1 µg/ml Trypsin: Sigma). In parallel control samples, Trypsin was inhibited with 50 µg/ml trypsin inhibitor (Soyabean; Gibco Invitrogen). Cells and protease were incubated at 37°C for 1 h with gentle shaking. Whole cells were harvested by centrifugation at 3,000×g for 5 min, and resuspended in SDS-PAGE loading buffer (400 µl/OD_600_ unit of bacteria).

### Cell fractionation

Induced cultures (500 ml) were washed with PBS three times and resuspended in 20 ml PBS. The OD_600 nm_ was normalised to 1.0 in PBS. To prepare the whole cell control, 1 ml was centrifuged at 6000×g for 5 min, and resuspended in 200 µl of SDS PAGE loading buffer [105]. To prepare the periplasmic and cytoplasmic fractions, 1 ml of the washed cells was centrifuged at 6000×g for 2 min at room temperature, and washed with 300 µl of 25 mM Tris pH 7.4 three times. The pellet was then resuspended in 50 µl of 25 mM Tris pH 7.4. and 1 µl of 0.1 M EDTA and 50 µl of 40% w/w sucrose in 25 mM Tris pH 7.4 were added. The sample was mixed gently at room temperature for 10 min. Subsequently, the sample was centrifuged and the pellet was resuspended in 100 µl of ice cold 0.5 mM Magnesium Sulphate. The sample was incubated on ice for 10 min, and centrifuged for 5 min at 13.000×g. The supernatant was taken as the periplasmic fraction. The pellet was resuspended in 600 µl of 10 mM Tris pH 7.4 plus 20 µg/mL (PMFS). The sample was frozen and thawed three times on dry ice. Following this, 19.9 µl, 1 M MgCl_2_ and 1.2 µl 1 mg/ml DNAase I were added. This was incubated at 37°C for 15 min. Next, the sample was centrifuged for 15 min at 13.000×g, and the supernatant contained the cytoplasmic fraction.

The rest of washed cells (18 ml) was centrifuged for 10 min at 13.000×g and resuspended in 3 ml 20 mM Tris pH 7.4 plus 1 mg DNAase I and 1 mg RNAase. The sample was passed through the French Press three times at 16000 lb/in, on ice. Next, the sample was centrifuged at 2000×g for 20 min at 4°C. The resultant supernatant was centrifuged again 27000×g for 40 min at 4°C. The pellet was resuspended in 200 µl 20 mM Tris pH 7.4 plus 0.7% (Sodium Lauryl Sarcosinate (SLS). The sample was incubated at 4°C for 25 min and centrifuged at 27000×g for 40 min at 4°C. Subsequently, the supernatant contained the inner membrane fraction and the pellet was resuspended in 200 µl 20 mM Tris pH 7.4 containing the outer membrane.

The samples (periplasmic, inner membrane, cytoplasmic and outer membrane fractions) were subjected to trichloroacetic acid (TCA) precipitation. The samples were supplemented to give a final concentration of 10% TCA, incubated on ice for 30 min, and centrifuged for 15 min at 13000×g. The supernatant was removed and 500 µl of ice cold acetone added. Following centrifuged for 5 min at 13000×g, the supernatants were discarded and pellets air dried for 15 min. Finally, the pellets were resuspended in 20 µl of 50 mM NaOH plus 180 µl SDS PAGE Loading Buffer [105].

### SDS PAGE and immunoblotting

Protein samples were prepared in loading buffer and boiled for 5 minutes before being subjected to SDS-PAGE or immunoblotting as previously described [105]. The mouse α-His monoclonal antibody (Novagen) was used at a concentration of 1∶2000, rabbit α-RpoS [113] was used at a concentration of 1∶10,000, rabbit α-IcsS was used at 1∶1,000 (kind gift from Emma Bouveret), rabbit α-LEP was used at 1∶5000 (kind gift from Vassilis Koronakis [114]), rabbit α-TolC at 1∶2000 (kind gift from Vassilis Koronakis [115]), rabbit α-AaaA was used at a concentration of 1∶1000 following preadsorption with a bacterial lysate. Binding was detected with secondary antibodies: α -mouse-HRP (Sigma, used at 1∶2000) and α-rabbit-HRP (Sigma, used at 1∶2000). Proteins recognized by the antibodies were revealed using an ECL detection kit (Pierce) and photographic film (Amersham) according to the manufacturer's instructions. Preadsorption of antisera was performed as described in [116]. Proteins were quantified by densitometry using ImageJ software (http://rsbweb.nih.gov/ij/). The protein band of interest on scanned images of SDS PAGE or Immunoblots was selected and the profile of density obtained. Gating to select the peak of interest was undertaken and the area underneath used as the relative density in the provided units. A matched area of background from the negative control was subtracted from this value. Where indicated, fold change was calculated by dividing the density of one protein band with that of the positive control.

### Confocal fluorescent microscopy

The instrument used was Zeiss LSM700, and all manipulations were performed in a humidifying chamber. Cells were fixed by mixing with an equal volume of 4% paraformaldehyde (4% v/v) and incubating for at least 60 min. Aliquots of the fixed bacteria were air dried onto a microscope slide and re-hydrated in two changes of freshly prepared Phosphate buffered saline (PBS). Following incubation in PBS containing 5% (w/v) bovine serine albumin (BSA) for 60 min, the fixed bacteria were incubated for 2 h with α-AaaA (1∶200 final concentration pre-absorbed sera in PBS-5% BSA). Following thrice washing in PBS, the cells were incubated for 2 h in donkey α-rabbit alexa fluor 680 conjugated secondary antibody (1∶400 in PBS, Invitrogen). Following three washes in PBS, the cells were incubated for 5 mins in FM1-43 (1∶250 in PBS). After mounting cover slips using fluorescent mountant (Sigma Flouromount f4680), slides were stored in the dark until imaging was undertaken on a Zeiss LSM 700. For the FM1-43 label excitation at 510 nm and emission at 626 nm was used, and for alexa fluor 680: excitation at 488 nm and emission at 702 nm. Zen software enabled images to be merged and viewed in either 2D or 3D.

### Tissue sectioning, staining, and microscopy

Skin infection sites were formalin-fixed and paraffin embedded. Sections (5 micron) were taken from a representative area and stained with hematoxylin and eosin [117].

### Mouse models

Mice were administered acute burn and chronic wounds as previously described [54]. Briefly for chronic wounds, mice were anesthetized, shaved and administered a dorsal, full-thickness, 1.5×1.5 cm surgical excision wound. The wounds were covered with a transparent, semipermeable polyurethane dressing (OPSITE, Smith & Nephew, Hull, England) which allowed for daily inspection of the wound, wound size determination, topical application of bacteria onto the wound, and protection from other contaminating bacteria. In addition, the OPSITE dressing acts as a mechanical barrier to wound contraction, physically holding the wound open and resulting in a slow-healing wound. A total of 10^4^ CFU PAO1 or the Δ*aaaA* mutant were injected under the dressing, on top of the wound. Mice were euthanized at 8 days post-infection and tissue from their wounds was harvested, weighed and homogenized in sterile PBS. Colonies were enumerated on LB agar to determine the CFU/g tissue.

## Supporting Information

Figure S1 **An in-frame deletion mutant of** ***aaaA*** **grows similarly to its parent in rich medium.** (**Panel A**) The cartoon indicates the strategy used to generate the Δ*aaaA* mutant. Primer positions are indicated as aaaAfa (a), aaaArb (b), aaaAfb (c), and aaaArc (d). Genomic DNA from the parental PAO1 and Δ*aaaA* mutant was digested with *Xmn*I, and hybridised to a probe directed against *aaaA*. The Southern blot shows the expected sizes of DNA were detected (wt: 4.8 kb; Δ*aaaA*: 2.9 kb). Migration of marker DNA fragments is indicated in kb on the left. (**Panel B**) PAO1 and the Δ*aaaA* mutant were grown in LB medium and the absorbance of the culture at 600 nm is shown plotted against time of growth.
(TIF)

Figure S2 **AaaA does not remove methionine or Leucine from** ***p*** **-nitroanilide.** **Panel A**. SGAP was incubated with either leucine-*p*-nitroanilide (solid circle) or arginine-*p*-nitroanilide (open circle) and the resultant changes in A_405 nm_ are shown against time compared with a buffer blank (crosses). **Panel B.** The *P. aeruginosa* Δ*aaaA* mutant (open circles) or WT PAO1 cells (closed circles) were grown in LB broth until OD_600 nm_ of 1.5, and then incubated with methionine-*p*-nitroanilide as described in materials and methods. Activities are compared against a growth media blank (crosses) **Panel C.** The *P. aeruginosa* Δ*aaaA* mutant (open circles) or WT PAO1 cells (closed circles) were treated as described in Panel B, except leucine-*p*-nitroanilide was used as a substrate. Activities are compared against a growth media blank (crosses). Error bars are+/−1 S.D. (n = 15). All measurements have been corrected for differential growth of bacteria by normalising to an OD_600 nm_ of 0.1.
(EPS)

Figure S3 **Insertion of MiniCTX does not significantly alter the growth profile of** ***P. aeruginosa*** **in LB.** Wild type *P. aeruginosa* PAO1 (closed triangles), the Δ*aaaA* mutant (open circles) and the Δ*aaaA* mutant with insertion of ctx::*aaaA* PAJL (closed circles) were inoculated at an OD of 0.06 in LB, and the growth over 18 h as monitored in the automated plate reader tecan at 612 nm is shown.
(EPS)
