# Supplementary figures and images for: A Novel Virulence Strategy for Pseudomonas aeruginosa Mediated by an Autotransporter with Arginine-Specific Aminopeptidase Activity
Source: PLoS Pathog. 2012 Aug 23;8(8):e1002854. doi: 10.1371/journal.ppat.1002854 (PMC3426542; doi:10.1371/journal.ppat.1002854)

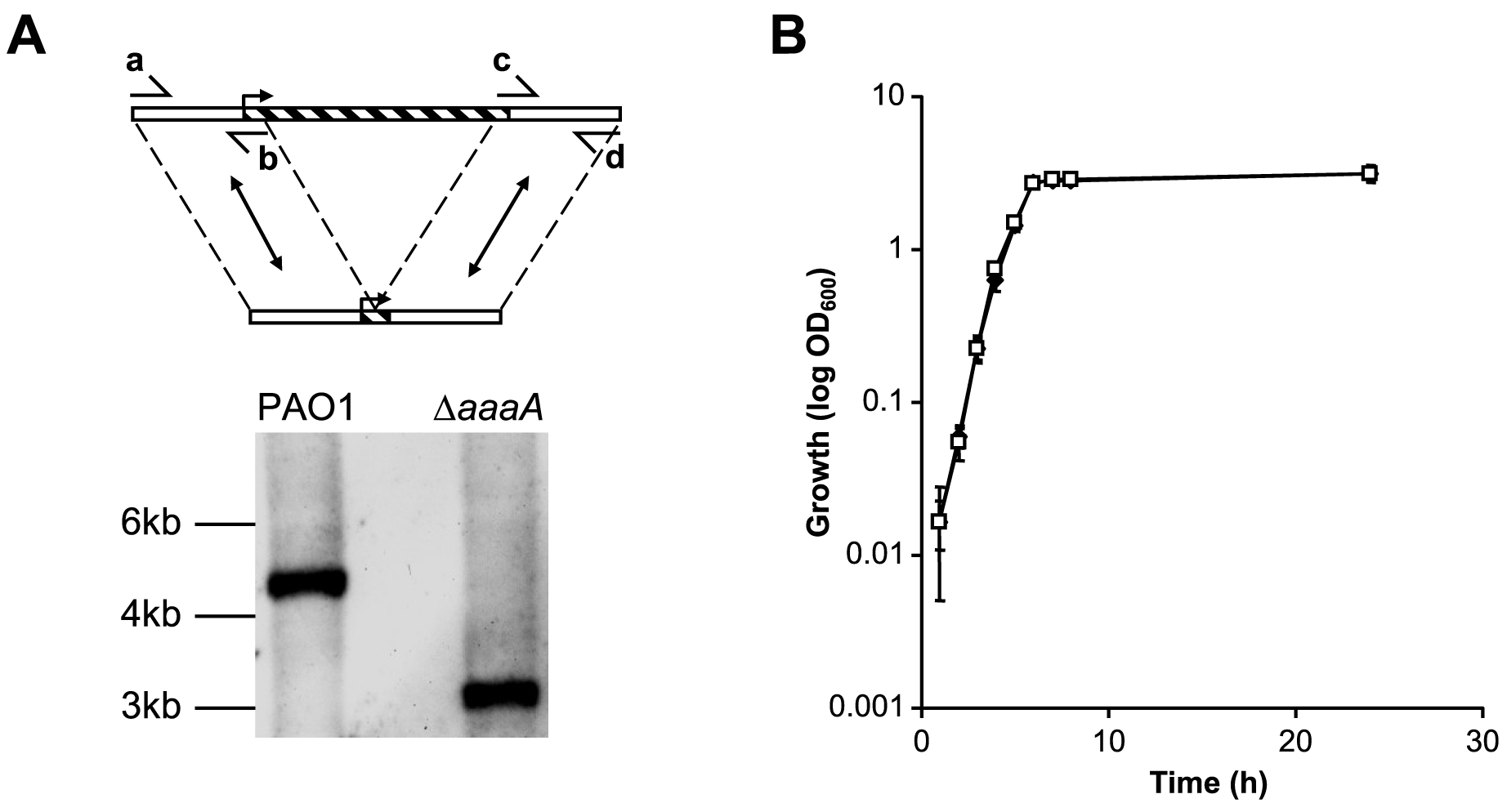

Supplement: Figure S1 — An in-frame deletion mutant of aaaA grows similarly to its parent in rich medium. (Panel A) The cartoon indicates the strategy used to generate the ΔaaaA mutant. Primer positions are indicated as aaaAfa (a), aaaArb (b), aaaAfb (c), and aaaArc (d). Genomic DNA from the parental PAO1 and ΔaaaA mutant was digested with XmnI, and hybridised to a probe directed against aaaA. The Southern blot shows the expected sizes of DNA were detected (wt: 4.8 kb; ΔaaaA: 2.9 kb). Migration of marker DNA fragments is indicated in kb on the left. (Panel B) PAO1 and the ΔaaaA mutant were grown in LB medium and the absorbance of the culture at 600 nm is shown plotted against time of growth. (TIF) [file ppat.1002854.s001.tif]
